# Proteomic profiling of metabolizing enzymes and transporters in 2 layers of Caucasian human skin

**DOI:** 10.1016/j.dmd.2026.100310

**Published:** 2026-04-29

**Authors:** Seun Seriki, Amin Rostami-Hodjegan, Stacey Warwood, Ming-Liang Tan, Eleftheria Tsakalozou, Jill Barber, Zubida M. Al-Majdoub

**Affiliations:** 1 Centre for Applied Pharmacokinetic Research, Division of Pharmacy and Optometry, School of Health Sciences, University of Manchester, Manchester, United Kingdom; 2 Certara Predictive Technologies, Certara Inc, Sheffield, United Kingdom; 3 Biological Mass Spectrometry Core Facility, University of Manchester, Manchester, United Kingdom; 4 Division of Quantitative Methods and Modeling, Office of Research and Standards, Office of Generic Drugs, Center for Drug Evaluation and Research, U.S. Food and Drug Administration, Silver Spring, Maryland

**Keywords:** Skin, Enzymes, Transporters, LC MS/MS, Dermis, Epidermis

## Abstract

Topical and transdermal drug and cosmetic development is advancing across industry; however, the efforts may in part be hindered by paucity of data on the fate of chemicals in human skin, which depend on protein-mediated transport and biotransformation of such chemicals. A label-free mass spectrometry-based proteomics approach was used to comprehensively quantify drug metabolizing enzymes and transporters in the fractionated epidermis and dermis of 17 healthy Caucasian human skin samples. Over 1000 proteins were identified for cytosolic and membrane components, and abundance were obtained using a modified HiN (high 3/2 ion intensity) approach (without using standards). Key findings included high interindividual variability in the expression of phase I and II enzymes (eg, aldehyde dehydrogenase 2, carboxylesterase 1, glutathione S-transferase P1, and glutathione peroxidase 3) and solute carrier transporters (eg, SLC25A5, SLC25A6). Notably, several metabolic enzymes, previously uncharacterized in human skin, were quantified in the membrane fractions, including glutathione S-transferases (GSTs) such as GSTM3 (12.2 pmol/mg protein) and GSTP1 (5.55 pmol/mg protein). Subcellular localization analysis revealed that many quantified proteins were associated with mitochondrial or membrane compartments, reinforcing the functional diversity and compartmentalized nature of dermal metabolism. Furthermore, strong correlations in enzyme and transporter expression were observed between the dermis and epidermis (Spearman’s *ρ* > 0.85). This dataset provides the most detailed quantification to date of drug metabolizing enzymes and transporters in human skin and offers critical input parameters for dermal physiologically based pharmacokinetic modeling applications. These quantitative insights improve the accuracy and clinical relevancy of in vitro in vivo extrapolations related to dermal drug metabolism and disposition for chemicals applied to the skin or those that come into contact with the skin inadvertently. Integration of this proteomic dataset into physiologically based pharmacokinetic frameworks will enhance the scientific reliability, applicability across product applications and regulatory acceptance of skin-based models for drug development and safety evaluation.

**Significance Statement:**

Understanding drug metabolism and transport in human skin is essential for predicting dermal absorption and safety. This study provides the most comprehensive proteomic dataset of metabolizing enzymes and transporters in epidermis and dermis, revealing high interindividual variability, previously unquantified enzymes, and strong layer correlations, supporting improved dermal physiologically based pharmacokinetic model development.

## Highlights

• Comprehensive proteomic profiling of epidermis and dermis from 17 human skin donors.
• Over 1000 cytosolic and membrane proteins quantified using label-free mass spectrometry.
• High interindividual variability observed in key metabolic enzymes and transporters.
• Previously uncharacterized skin enzymes, including carboxylesterase 1 and glutathione S-transferase isoforms, were quantified.
• Strong dermis-epidermis correlations (Spearman’s *ρ* > 0.85).
• Dataset provides critical input parameters for improving dermal physiologically based pharmacokinetic and in vitro in vivo extrapolation predictions.

## Graphical abstract

**Figure:**
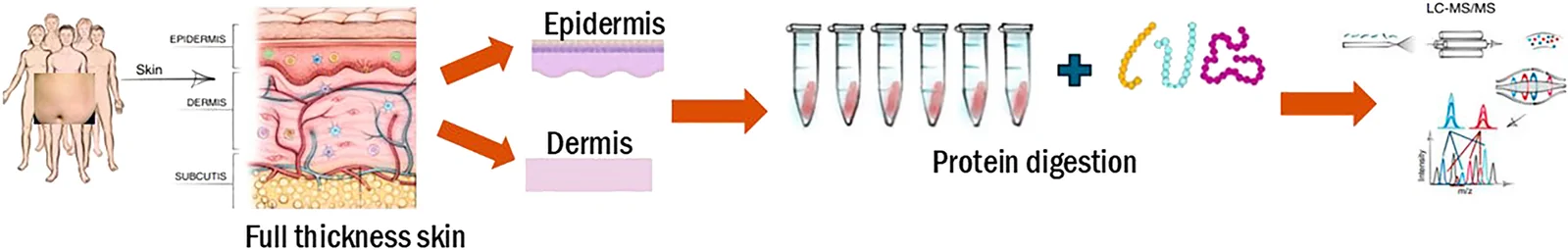

## 1 Introduction

The use of human-derived in vitro systems requires in vitro in vivo extrapolation that relies on knowledge of scaling factors from in vitro systems to the in vivo conditions.^1^ Many of these pieces are not fully in place. This gap is particularly pressing for topical and transdermal products, where skin-specific parameters for protein-mediated transport and biotransformation are needed to support in vitro in vivo extrapolations. Against this backdrop, the global demand for topical drug delivery has seen a significant rise within the pharmaceutical and dermatological sectors, with the market projected to grow from approximately $109 billion to $177 billion between 2023 and 2030.^2^ These types of therapies involve applying medications directly onto the surface of the skin or via transdermal administration,^3^^,^^4^ where active compounds can penetrate the skin’s layers to exert localized therapeutic effects, and in some cases, produce systemic effects.^5^ A key advantage of this route is the circumvention of hepatic first-pass metabolism, which can otherwise limit drug absorption and lower bioavailability. Human skin is composed of 3 main layers; epidermis, dermis, and hypodermis with the outermost layer (epidermis) primarily made up of keratinocytes that form a protective barrier and regulate immune responses.^6^ This complex structure, populated by specialized cells such as Langerhans cells, plays a critical role not only in shielding the body from pathogens but also in modulating drug absorption, metabolism, and immune-mediated reactions, making it highly relevant for transdermal drug delivery studies.^7^ Few studies^8^, ^9^, ^10^, ^11^ are available on drug metabolism in human skin, and most have focused on enzymes and pathways already established in liver and intestines.^12^^,^^13^ Nevertheless, evidence is mounting for metabolically competent enzymes in skin.^8^^,^^11^^,^^14^ Drug metabolizing enzymes can detoxify xenobiotics but may also generate reactive or toxic intermediates that provoke adverse skin reactions.^15^^,^^16^ For instance, diclofenac undergoes hydrolysis by esterases in skin, forming metabolites that influence efficacy and clearance.^17^ Topical and transdermal applications may be intended for either local or systemic exposure; diclofenac^17^ and nicotine^18^ exemplify compounds designed for local anti-inflammatory and systemic delivery, respectively, and therefore require sufficient permeation across the skin barrier. In contrast, for compounds such as insecticides and UV filter chemicals, skin barrier function and metabolic activity are desirable, limiting systemic exposure through local detoxification. In these contexts, cutaneous metabolism can act as a first-pass protective mechanism. Quantitative characterization of skin-specific enzymes and transporters is therefore essential for predicting dermal drug disposition and systemic exposure. Recent proteomic and transcriptomic studies have reported cytochrome P450 1A1 (CYP1A1), CYP1B1, CYP2E1, and CYP3A4 in both epidermis and dermis, suggesting a role in the metabolism of topically applied drugs and environmental contaminants.^11^^,^^19^ However, the available data are limited, by small cohorts or by lack of a clear separation between dermal and epidermal layers. In addition to phase II enzymes, hydrolases and dehydrogenases may also be present based on reverse-transcription polymerase chain reaction, immunohistochemistry, and catalytic assays.^20^, ^21^, ^22^, ^23^, ^24^, ^25^ Although cutaneous metabolism may contribute to localized effects, systemically circulating drugs are predominantly metabolized hepatically.^12^^,^^26^^,^^27^ The skin functions primarily as a barrier, with its stratum corneum restricting both water loss and xenobiotic entry. Absorption and distribution are largely governed by passive diffusion, although membrane transporters, selectively modulate drug movement across skin layers, influencing local retention and potential systemic exposure.^28^ Beyond barrier and transport mechanisms, skin metabolism encompasses broader proteomic networks, including antioxidant enzymes, proteases, and peptidases that modulate oxidative stress, facilitate structural remodeling, and regulate immune responses functions increasingly relevant for peptide therapeutics and cosmetic formulations.^29^^,^^30^ Topical formulations offer advantages for patients (eg, reduced gut effects and potentially more controllable systemic concentrations), but only molecules that penetrate the barrier sufficiently are viable candidates. Entry to the bloodstream relies on suitable transport systems, either passive or mediated by transporters. Thus, a quantitative understanding of these transporters is required to assess transdermal suitability. Mass spectrometry-based quantitative proteomics has emerged as a powerful strategy for investigating protein abundance in complex tissues, including skin,^11^^,^^12^^,^^31^ however, high lipid content, limited protein solubility, and extensive crosslinking poses challenges for protein extraction and enzymatic digestion, which are essential for liquid chromatography–tandem mass spectrometry (LC-MS/MS) workflows.

In this study, we aimed to systematically quantify enzymes, transporters, and other relevant proteins across 2 layers of human skin (epidermis and dermis) in 17 healthy Caucasian donors using label-free LC-MS/MS proteomic analysis. The proteomic dataset generated in this study has the potential to provide parameters for dermal physiologically based pharmacokinetic (PBPK) models, which are increasingly used in topical drug development.^32^ Furthermore, this approach aligns with international initiatives on new approach methodologies for drug and cosmetic product development^33^^,^^34^ aimed at reducing animal testing by enabling the development of validated, nonclinical methods for predicting human skin exposure.

## 2 Experimental section

### 2.1 Study population, ethics, and sample handling

Seventeen Caucasian adult skin samples (16 women, 24–69 years; 1 man, 43 years) were obtained as surplus tissue from routine abdominoplasty at the University’s teaching hospital (University of Bradford). Collection was approved by the Research Ethics Committee (07/H1306/98+5), and samples were fresh-frozen and stored at −80 °C until processing. Samples are coded “C” followed by the donor’s age (eg, C24); duplicate agesf are distinguished by “a/b” (2 donors aged 49 years). Full demographics are provided in Supplemental Table 1.

### 2.2 Chemicals and reagents

Unless otherwise specified, all chemicals were obtained from Sigma-Aldrich at the highest purity available. The membrane extraction kit (Minute) and Invent kit (Biotechnologies, Inc) were used for plasma membrane protein isolation and cell fractionation kit, respectively. The 50X protease inhibitor cocktail was supplied by Promega (UK Ltd), whereas sequencing-grade modified trypsin was purchased from Roche Diagnostics GmbH. The QconCAT standards (TransCAT2, MetCAT, and NuncCAT) were internally designed, synthesized, and purified by PolyQuant GmbH, with the methodology and applications extensively described in previous studies.^35^ Additionally, non-naturally occurring peptides used for QconCAT quantification (not described in this publication) were procured from Cambridge Peptides, following established protocols for their integration into quantitative proteomic workflows.^31^^,^^36^

### 2.3 Tissue sample preparation

**Fig. 1:**
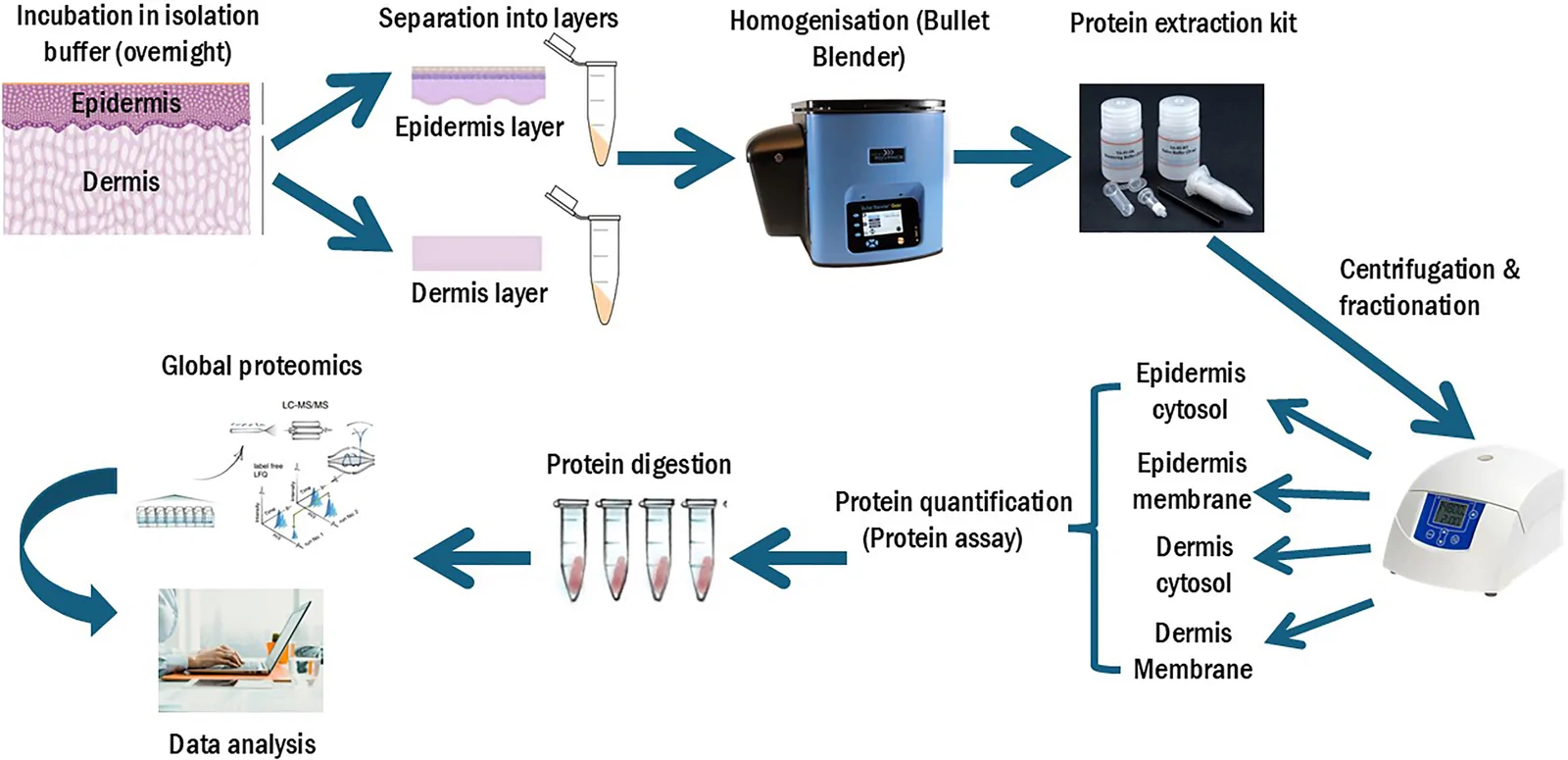
Schematic overview of skin sample processing and proteomic workflow. Human abdominal skin was separated into epidermis and dermis, followed by subcellular fractionation into cytosolic (EC, DC) and membrane (EM, DM) fractions. Each fraction underwent detergent removal, protein digestion, and LC-MS/MS analysis.

Approximately 0.3 to 0.6 g of skin was manually excised from human skin specimen using a surgical scalpel and incubated in a buffer containing 20 mM EDTA in high-pressure liquid chromatography water for at least 4 hours or overnight.^37^ After incubation, the skin samples were washed thoroughly with PBS buffer (Thermo Fisher) (pH 7.4) to remove residual incubation buffer. The epidermis and dermis were manually separated, and finely minced using a scalpel, then transferred into individual Eppendorf tubes. The epidermis fraction was combined with 1.8 mL of buffer A (Invent Biotechnologies, Inc) along with half a spoonful (0.5 g) of 3.2 mm steel beads. A 35.2 *μ*L aliquot of protease inhibitor (from a 50× stock reconstituted in 1 mL ethanol) was also included. For the dermis fraction, 0.94 mL and 18.8 *μ*L of buffer A and PI, respectively were added with a full spoonful of 3.2 mm steel beads. Each layer was homogenized using the Bullet Blender 24 Gold (NAI-BB24-AU, Next Advance), precooled using dry ice at maximum speed (setting 12) for 10 minutes and repeated 5 times to ensure a consistent homogenate was obtained. The homogenate from each layer was centrifuged using a microcentrifuge (Fisher Scientific) for 1 minute (1000 *g*) to remove debris. The resulting supernatant was then centrifuged at 4 °C for 45 minutes at 16,000 *g* to separate cytosolic and membrane fractions. The resulting supernatant (cytosolic fraction) was aliquoted and stored. The pellet (membrane fraction) was resuspended in 500 *μ*L buffer B (the Minute membrane extraction kit), followed by centrifugation at 7800 *g* for 5 minutes at 4 °C. The final membrane protein pellet was resuspended in 200 *μ*L storage buffer (0.5 M potassium carbonate [K_2_CO_3_], pH 7.0). All fractions were cleaned using detergent removal spin columns (Thermo Scientific), following the manufacturer’s protocol. The cleaned fractions were then aliquoted, snap-frozen, and stored at −80 °C until further analysis. Total protein concentration in both fractions (cytosolic and membrane) was measured using BCA protein assay kit (reducing agent compatible; Thermo Scientific Pierce) according to the manufacturer’s instructions. Protein concentrations were subsequently measured using Pierce BCA Protein Assay Kits. The overall experimental workflow, including epidermis–dermis separation, preparation of cytosolic and membrane fractions, and downstream LC-MS/MS analysis, is summarized schematically in Fig. 1.

### 2.4 Preparation of skin fractions for proteomic analysis

The cytosolic and microsomal fractions from each layer (epidermis and dermis) were processed using filter-aided sample preparation, as described in previous studies.^12^^,^^26^^,^^38^ Fifty micrograms of total protein from each fraction was processed. Each fraction was spiked with stable isotope-labeled standards; MetCAT (50 *μ*L from 1:10 dilution), TransCAT (10 *μ*L from 1:100 dilution), and NuncCAT (10 *μ*L from 1:100 dilution)^39^ before undergoing enzymatic digestion. To enable label-free quantification of the whole proteome, bovine serum albumin (BSA) was used as an internal standard. A 6 mg/mL stock solution of BSA was first diluted 1:2000, and 5 *μ*L (0.015 *μ*g) of the diluted solution was then added to each fraction. Total protein concentrations were determined before solubilization using the BCA assay (Thermo Fisher Scientific) following the manufacturer’s protocol. For downstream processing, 50 *μ*g of protein per sample was used. Proteins were then solubilized with sodium deoxycholate at a final concentration of 10% (w/v) in 8 M urea and 100 mM Tris-HCl, pH 8.5, and disulfide bonds were reduced using 100 mM 1,4-dithiothreitol. After brief vortexing and centrifugation for 1 minute, the samples were left at room temperature (RT) for 10 minutes, followed by incubation at 56 °C for 30 minutes. Centrifugal filter units (Amicon Ultra, Merck; MWCO 10,000) were washed twice with 200 *μ*L of 0.1 M Tris-HCl, pH 8.5 and centrifuged at 14,000 *g*. Samples were transferred to the filters and centrifuged at 14,000 *g* for 20 minutes at RT. This was followed by 4 washing steps using 200 *μ*L of 8 M urea in 0.1 M Tris-HCl, pH 8.5, with each wash followed by centrifugation at 14,000 *g* for 20 minutes at RT. The sample was alkylated with 100 *μ*L of 50 mM iodoacetamide, mixed in a thermomixer at 600 rpm for 1 minute at RT, and then incubated in the dark for 30 minutes. The samples were then centrifuged at 14,000 *g* for 10 minutes at RT, followed by 2 washes with 200 *μ*L of 8 M urea, each involving centrifugation at 14,000 *g* for 20 minutes at RT. An additional washing step was then performed. Buffer exchange was then carried out to 1 M urea in 50 mM ammonium bicarbonate, pH 8.0. Proteolytic digestion used Lys C (2% w/v at 30 °C for 3 hours), followed by trypsin (4% w/w at 37 °C for 17 hours).^40^ Peptides were recovered by centrifugation at 14,000 *g* for 20 minute. Recovered peptides were desalted using Pierce C18 spin columns (Thermo Scientific), according to the manufacturer’s protocol, and lyophilized by speed vacuum centrifugation (Eppendorf).

### 2.5 Liquid chromatography analysis sample preparation

The samples were reconstituted in 30 *μ*L of loading buffer (3% acetonitrile and 0.1% formic acid) and spiked with unlabeled peptide standards; GVNDNEEGFFSAR, VGFAFDLPWGSIK, and GISNEGQNASIK for MetCAT quantification at known amount (67 fmol); AEGVNDNEEGFFSAR and TEGVNDNEEGFFSAR for NuncCAT quantification at known amount (42 pmol); and VGFLPDGVIK and EGVNDNEEGFFSAR for TransCAT quantification at known amount (22 fmol) and the mixture was transferred into sample vials.

### 2.6 Liquid chromatography and mass spectrometry workflow

Peptides from each fraction were analyzed by LC-MS/MS using a Thermo RSLC system consisting of a NCP3200RS nano pump, WPS3000TPS autosampler, and TCC3000RS column oven configured with buffer A as 0.1% formic acid in water and buffer B as 0.1% formic acid in acetonitrile. An injection volume of 2 *μ*L was loaded into the end of a 5 *μ*L loop and reversed flushed on to the analytical column (Waters nanoEase M*/Z* Peptide CSH C18 Column, 130 Å, 1.7 *μ*m, 75 *μ*m × 250 mm) kept at 35 °C at a flow rate of 300 nL/min with an initial pulse of 500 nL/min for 0.1 minute to rapidly repressurize the column. The separation consisted of a multistage gradient of 1% B to 6% B over 3 minutes, 6% B to 18% B over 67 minutes, 18% B to 29% B over 9 minutes, and 29% B to 65% B over 1 minute before washing for 6 minutes at 65% B and dropping down to 2% B in 1 minute. The complete method time was 120 minutes. The analytical column was connected to a Thermo Exploris 480 mass spectrometry system via a Thermo nanospray Flex Ion source via a 20 *μ*m ID fused silica capillary. The capillary was connected to a fused silica spray tip with an outer diameter of 360 *μ*m, an inner diameter of 20 *μ*m, a tip orifice of 10 *μ*m, and a length of 63.5 mm (New Objective Silica Tip FS360–20–10-N-20–6.35CT) via a butt-to-butt connection in a steel union using a custom made gold frit (Agar Scientific AGG2440A) to provide the electrical connection. The nanospray voltage was set at 1900 V and the ion transfer tube temperature set to 275 °C.

### 2.7 Protein identification and quantification

Protein and peptide identification and quantification were performed using Progenesis v4.0 (Nonlinear Dynamics) and Mascot, version 3.0 (Matrix Science). Tandem mass spectra were searched against the UniProt human proteome database (May 2017, 71,599 entries).^41^ Within Mascot, the precursor mass tolerance was set to 2 ppm, and the fragment mass tolerance was 0.02 Da. Cysteine carbamidomethylation was set as a fixed modification, whereas oxidation of methionine was included as a variable modification. Trypsin/P was selected as the proteolytic enzyme, allowing for one missed cleavage. After protein identification, the resulting list was imported back into Progenesis for quantification. Protein abundance was calculated using a modified HiN approach, designed to accommodate the challenges associated with proteomic analysis of complex tissue such as human skin. In the standard HiN method, quantification is based on unique peptides and normalized against a reference protein, typically BSA. In our adaptation, we introduced 2 key modifications:

1. Normalization was achieved without the use of a standard. Although we developed this approach (which has similarities to the total protein approach method) for use with historical samples where no standard had been added, it has also proved useful where, as in this case, there is evidence of poor mixing of standard and sample (Izat et al, unpublished data). This tends to occur in fibrous samples, such as tumor, and, of course, skin. Instead of using an external standard, the weighted average molecular weight of the proteins in the sample is calculated and used to determine the total pmol/mg protein, against which individual proteins are normalized.
2. Inclusion of nonunique peptides: To permit the quantification of closely related and polymorphic proteins, we included nonunique peptides by proportionally distributing the intensity of shared peptides among all matched proteins. This approach maintains quantification accuracy while expanding the protein coverage beyond what is possible with unique peptides alone.

Protein abundances were derived using the sum of the top 3/2 most intense peptides (Hi3/Hi2) per protein. To avoid bias from standard peptides, assignments based solely on QconCAT-derived peptides were excluded from final analyses. All protein abundance values were reported as picomole of protein per milligram of total protein. For the purposes of this study, a transporter or enzyme was considered quantified when at least 2 peptides were detected with sufficient intensity to contribute to a Hi2/Hi3 estimate; proteins detected with only 1 peptide or with intensities below this threshold were classified as detected but below the limit of quantification (BLOQ). Nondetection in the summary tables therefore reflects either absence of any confidently assigned peptide or presence only at BLOQ levels under these criteria.

Formal validation of analytical sensitivity and precision using independent low abundance membrane transporter standards was not feasible because of low signal intensities observed for membrane proteins. Instead, analytical robustness was assessed through internal consistency checks, including reproducibility of peptide detection across donors, concordant abundance patterns between epidermal and dermal layers, and agreement with known subcellular localization. The consistent detection of mitochondrial solute carrier transporters across samples, alongside the systematic classification of single peptide detections as BLOQ, supports the reliability of the reported quantitative trends despite the recognized limitations in absolute sensitivity for low abundance membrane proteins. Subcellular fraction enrichment was assessed based on proteomic localization patterns rather than classical marker-based validation. Cytosolic fractions were enriched in soluble metabolic enzymes, whereas membrane fractions showed consistent enrichment of membrane-associated proteins, particularly solute carrier transporters.

### 2.8 Statistical data analysis

Statistical analysis was conducted with GraphPad Prism 9.3.1. The data were assessed for normality and statistical significance was determined using the Mann-Whitney test. Changes were deemed statistically significant if the *P* value was below .05.

## 3 Results

### 3.1 Overview of LC-MS/MS results

The skin samples used in these experiments were derived from human abdomen, specifically abdominoplasty. They were therefore difficult to handle because of fat and because the skin itself is made up largely of insoluble material. The membrane samples were therefore weak, with typically total intensity 10^9^ as analyzed by Progenesis, compared with 10^10^ in the cytosolic samples. The total number of peptides detected was small for all samples – below 10000. This compares with 20,000 to 30,000 for the majority of liver microsome samples analyzed in this laboratory using the same instrumentation.^26^^,^^42^^,^^43^ Supplemental Table 2 summarizes the total intensities and peptide numbers for each sample.

### 3.2 Detection and evaluation of anomalous peptides

Razors for each fraction were prepared as described previously,^12^ revealing the presence of anomalously abundant peptides in all fractions. These are shown in Supplemental Table 3. By far the most prominent of these is GITLSVRP, assigned to the C-terminus of T cell receptor *α* joining 56,^44^ a comparatively poorly annotated 21-residue fragment. It has been observed in human liver membrane fractions also.^26^ Basic local alignment search tool searches were carried out for this peptide and for all possible variants, where L replaces I and vice-versa. Further hits in humans were all variants of T cell receptor *α* or *β*.

FQIAQVVR is found in the 300-residue protein 7-methylguanosine phosphate-specific 5′-nucleotidase, and is fully annotated. Similarly, PEYLVSGIRTPPVR is found in phosphatase and actin regulator 3, which has 559 residues. LVTSELK is found in a comparatively poorly annotated 62-residue fragment of diacylglycerol kinase eta, APECGAPALPR in the putative uncharacterized 130-residue protein encoded by LINC00472, and MAAPALK by *α*-mannosidase 2C1, which has over 1000 residues. We would expect to see other peptides from these proteins if the full protein were expressed at high levels in the skin. All these 6 peptides are automatically ignored in a HiN quantification. The present investigation is focused on drug metabolizing enzymes and transporters. Thus, IGSTPVLVLSR, assigned uniquely to CYP1A2, requires explanation (Supplemental Table 3). CYP1A2 (Supplemental Table 3) is annotated to the endoplasmic reticulum membrane^45^ and would not be expected to accumulate preferentially in the dermis and epidermis cytosol. It has a molecular mass in excess of 57 kDa so if the entire protein were present, other peptides would be expected. In fact, the only other peptides detected are YLPNPALQR and ASGNLIPQEK. These are both present in labeled form in the QconCAT standards (specifically the Fusion-MetCAT), as is IGSTPVLVLSR (found in the NuncCAT).^39^ Unlabeled isotopomers are always present at low level in these constructs and the amounts of unlabeled peptide detected are consistent with their being derived from the QconCATs. Supplemental Table 4 shows the total intensity of unlabeled peptide as a percentage of the labeled peptide detected. In the cytosolic fractions, the single peptide IGSTPVLVLSR appears, by contrast at 144% (epidermis) and 277% (dermis) (Supplemental Table 3) of the QconCAT standard. We have no complete explanation for this, but note that IGSTPVLVLSR appears at position 80, close to the N-terminus of CYP1A2 and may therefore derive from a truncated version of CYP1A2 and accumulate in the cytosol.

### 3.3 HiN analysis of protein abundances

The HiN method^46^ of global quantification normally relies upon using unique peptides only and on comparing intensities with a standard, typically BSA. We have made 2 modifications to this protocol. Firstly, we have taken advantage of the pmol/mg units (pmol of a protein against mg total protein) used normally in pharmacological measurements; if the weighted average molecular weight of proteins in a sample can be calculated, these units preclude the need for a standard (Izat et al, unpublished). This is very helpful for difficult samples in which mixing of a standard with the sample is often poor, leading to underestimates of sample proteins. Secondly, the exclusive use of unique peptides makes it difficult to quantify similar proteins, especially polymorphic proteins. We have previously described how a peptide’s total intensity may be divided among the proteins that contain that peptide,^12^ and this allows the use of nonunique peptides in quantification without serious risk to accuracy. By using a modified Hi3 quantification wherever possible and a Hi2 quantification when only 2 peptides are seen, we constructed HiN abundances in the skin for the top 10 proteins in each fraction, as shown in Table 1.

**Table 1:** Protein content of 4 fractions of Caucasian human skin (*n* = 17), expressed as pmol/mg protein

Epidermis Membrane – 1033 Proteins – Median WAMW 68118 (Range 43138–128552 Da)		Dermis Membrane – 1192 Proteins – Median WAMW 78776 (Range 43187–134498 Da)		Epidermis Cytosol – 1513 Proteins – Median WAMW 68879 (Range 54985–133716)		Dermis Cytosol – 1375 Proteins – Median WAMW 74733 (Range 59766–126392)	
Most Abundant Proteins	Median (Range) pmol/mg	Most Abundant Proteins	Median (Range) pmol/mg	Most Abundant Proteins	Median (Range) pmol/mg	Most Abundant Proteins	Median (Range) pmol/mg
TRY1	2030 (84–13k)	TRY1	1045 (0–17k)	ALBU	6137 (40–11k)	ALBU	6429 (2021–10k)
ALBU	1867 (110–6679)	CO1A1	513 (94–1394)	CO1A1	43 (8–3630)	IGKC	693 (173–1370)
CO1A1	417 (38–1626)	ALBU	1299 (219–3758)	CO1A2	12 (2–2399)	IGHG1	527 (178–1398)
K2C1	339 (79–1034)	CO3A1	308 (69–1626)	K1C10	7.6 (1–25.6)	IGHG2	430 (100–1306)
CO3A1	183 (63–507)	K2C1	173 (38–2090)	IGKC	268 (8–1871)	PAR14	425 (0–1260)
CO1A2	166 (14–784)	PGS2	421 (41–2187)	IGHG1	309 (14–1912)	TRFE	242 (165–936)
K1C10	208 (65–839)	GSHB	200 (0–1094)	CO4A1	9 (0.95–930)	LUM	94 (23–385)
LUM	101 (39–815)	CO1A2	172 (46–648)	IGHG2	124 (0.62–902)	FLNA	19 (6–1113)
IGKC	225 (24–587)	K1C10	105 (26–1236)	K2C1	12 (4–40)	PGS2	111 (30–570)
PGS2	82 (26–566)	LUM	288 (30–599)	TRFE	289 (2–805)	IGLL5	114 (36–844)

Full details are given in Supplemental Table 5.

Table 1 shows the top 10 proteins by mean abundance for each fraction. As expected, collagens abound (CO1A1, CO1A2, and CO3A1), especially in the membrane fractions, and keratins type 1 (K2C1) and type 2 (K1C10) are strongly present in the membrane fractions. Despite extensive washing of samples, albumin (ALBU) is strongly present. Immunoglobulins (IGKC and IGHG1) are very strongly represented in the most abundant proteins. Decorin (PGS2) is very abundant in dermis membrane fraction. It is another extracellular matrix protein, which binds to collagen. Lumican (LUM), similarly is an extracellular matrix protein, and Filamin-A (FLNA) is a cytoskeleton protein. Serotransferrin (TRFE) is responsible for iron transport into cells and is abundant in both epidermal and dermal cytosol. It is abundant in every sample of dermal cytosol, with a big scatter in epidermis. Protein mono-ADP-ribosyltransferase (PARP14) is found in very varying amounts in the dermis cytosol and is responsible for ribosylation of glutamate residues. Glutathione synthetase (GSHB) is only quantified in dermis membrane (Table 1). The extremely high abundance of trypsin 1 in the membrane fractions is notable and raises questions about its potential function in the skin. There are 4 peptides attributed to this protein, and all are found in all samples. However, these peptides are also found in porcine trypsin, which is used for proteolysis of these samples. Autolysis can normally be ruled out because the commercial trypsin is modified at all the lysine residues, and all these peptides are lysine terminated. Thus, the default assumption is that human trypsin is genuinely abundant in the membrane fractions. Studies have shown that trypsin 1 promotes eumelanin synthesis in melanocytes and contributes to melanoma progression by sequestering the tumor suppressor mRNA-16.^47^ In addition, trypsin 1 elevated mRNA levels in skin metastases are linked to poor prognosis, making it a potential therapeutic target.^48^ The Supplemental Table 5 shows in detail the abundances of these proteins in individual samples.

### 3.4 Drug metabolism enzymes and transporters in Caucasian human skin

The main focus of this work was to understand the potential for drug transport and metabolism in the human skin and therefore to quantify known transporters and enzymes in the 4 fractions tested here. Six hundred eighteen proteins have been identified as drug metabolizing enzymes, ATP-binding cassette (ABC) or solute carrier (SLC) transporters (collectively, drug metabolizing enzymes transporters) in humans. The analysis was performed separately on both dermis and epidermis in the microsomal and cytosolic fractions, and the resulting data were then combined.

### 3.5 Impact of QconCAT standards on peptide availability and quantification

All skin samples contained QconCAT standards for a number of these proteins and the presence of residual unlabeled peptides reduced the number of peptides available for quantification (peptides used in the standards were rejected). The quantification of these proteins (marked with an asterisk) therefore represents a minimum estimate.

### 3.6 ATP-binding cassette transporters

There was no convincing evidence of the presence of ABC transporters in any of the skin samples, in any of the fractions. Occasionally, single ABC-derived peptides were observed but fell BLOQ and are therefore reported as not quantified rather than truly absent.

### 3.7 Solute carrier transporters

In common with ABC transporters, SLC transporters (Table 2) accumulate in the membrane fractions of cells.^12^ Some cytosolic samples display small numbers of SLC transporter peptides at low level, but these are almost certainly either artifacts of sample preparation or the products of cellular proteolysis. Consistent with our criteria for quantification, such single, low-intensity SLC peptide signals were treated as BLOQ and not counted as quantified transporter expression in cytosolic fractions. The presence of human trypsin in skin samples makes the accumulation of tryptic peptides in unexpected fractions of cells more likely. The SLC peptides found in the cytosol are shown in Supplemental Table 6. ADT2 (SLC25A5) and ADT3 (SLC25A6) are ADP/ATP translocases found in the mitochondrial inner membrane. MPCP (SLC25A3) is found in the same membrane and transports copper or phosphate ions into the mitochondrial matrix. Mitochondria are not rigorously separated from cytosol in our preparation. S12A2 (SLC12A2) is a cation chloride cotransporter found in the basolateral cell membrane of many tissues.^49^ These 3 transporters were also found in the membrane fractions, where several other SLC transporters were also identified.

**Table 2:** SLC transporters found in the membrane fractions of Caucasian human skin (*n* = 17)

SLC Transporter	Number of Peptides		Abundance (Epidermis Membrane) Mean (pmol/mg) and CV (%)		Abundance (Dermis Membrane) Mean (pmol/mg) and CV (%)	
	Epidermis	Dermis				
ADT1	7	7	1.7	114	0.63	136
ADT2	11	11	8.2	117	7.1	126
ADT3	10	9	0.77	212	1	152
B3AT	5	10	0.04	187	0.63	184
CMC1	2	3	0.02	206	0.04	203
CTL1	—	2	—	—	0.01	187
CTL2	—	3	—	—	0.04	137
GTR1	—	3	—	—	0.1	118
M2OM	3	3	0.03	211	0.03	143
MPCP	6	7	1.3	90	1.6	174
SCMC1	2	3	0.03	197	0.08	157
S12A2	7	3	0.52	131	0.24	198
S35F3	—	2	—	—	0.08	117
TXTP	2	4	0.03	197	0.02	125

—, not detected.

Table 2 shows that 10 SLC transporters were found in the epidermis membrane; these and an additional 4 were found in the dermis membrane. Thus the 2 fractions are very similar. Samples C47, C49a, C52, and C54 had very low levels of transporters in both dermis and epidermis (Supplemental Table 7). Although these donors form quite a tight cluster by age (47–54 years), significant levels of transporter are found in samples from both younger and older donors.

### 3.8 Correlation of transporter protein abundances between epidermal and dermal membrane fractions

**Fig. 2:**
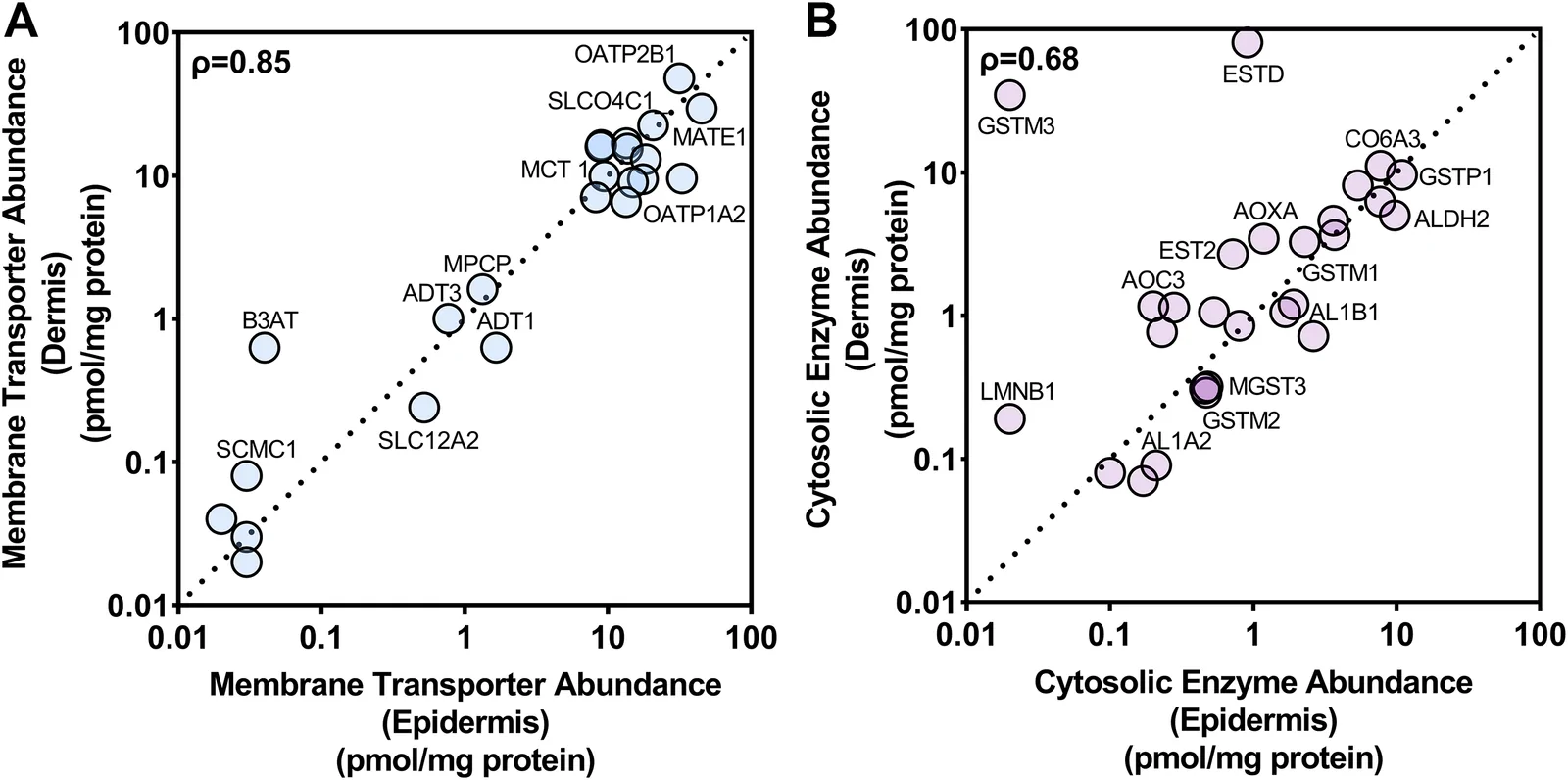
Correlation of protein abundance between epidermis and dermis in human skin. (A) Correlation of transporter protein abundance (pmol/mg protein, log scale) between epidermal and dermal membrane fractions. Each dot represents an individual protein. The dotted line represents the line of identity (*y* = *x*). A strong positive correlation was observed (Spearman’s *ρ* = 0.85), indicating overall similarity in protein expression profiles between the 2 skin layers. (B) Correlation of cytosolic enzyme abundance (pmol/mg protein) between the epidermis and dermis in 17 human skin samples. Each point represents one protein; the dotted line indicates the line of identity *(y = x)*. A strong positive correlation was also observed (Spearman’s *ρ* = 0.68).

A strong positive correlation (Fig. 2A) was observed between the 2 skin layers (Spearman’s *ρ* = 0.85), indicating a high degree of similarity in membrane transporter expression profiles. This suggests that, despite the structural and functional differences between the dermis and epidermis, transporter abundance is largely conserved across both skin layers.

### 3.9 Total expression of SLC transporters in both skin layers

Quantitative proteomic analysis of SLC transporters revealed substantial interindividual variability across the 17 samples analyzed. Among the transporters, ADT1/SLC25A4 and ADT2/SLC25A5 showed the highest mean expression levels (15.4 ± 15.3 pmol/mg protein and 2.95 ± 3.25 pmol/mg protein, respectively), suggesting a prominent role in mitochondrial transport processes. In contrast, transporters such as CTL1/SLC44A1, CTL2/SLC44A2, and S35F3/SLC35F3 exhibited very low expression levels, all below 0.1 on average, indicating potentially limited or highly regulated expression in the sampled tissue. High coefficients of variation were observed in B3AT/SLC4A1 (174%), S2512/SLC25A12 (157%), and CTL1/SLC44A1 (187%), reflecting considerable heterogeneity in transporter abundance, which may correspond to differences in metabolic or cellular demands across individuals. Several transporters, including SLC25A1, SLC25A3, and SLC25A6, demonstrated more consistent expression patterns, with coefficients of variations (CVs) ranging from ∼100%–115%. These findings underscore the differential regulation of SLC transporter proteins, which could influence the intracellular trafficking of ions, metabolites, or drugs, particularly in tissues with high energy requirements or specialized transport functions.

### 3.10 Total expression of enzymes in both skin layers

A range of non-CYP drug- metabolizing enzymes were quantified in human skin, including those involved in oxidation, reduction, and hydrolysis. These enzymes contribute to phase I metabolic activity and highlight the skin’s capacity for xenobiotic and prodrug metabolism beyond classical hepatic pathways.

### 3.11 Total expression and variability of phase I (non-CYP) enzymes in both layers of human skin

**Fig. 3:**
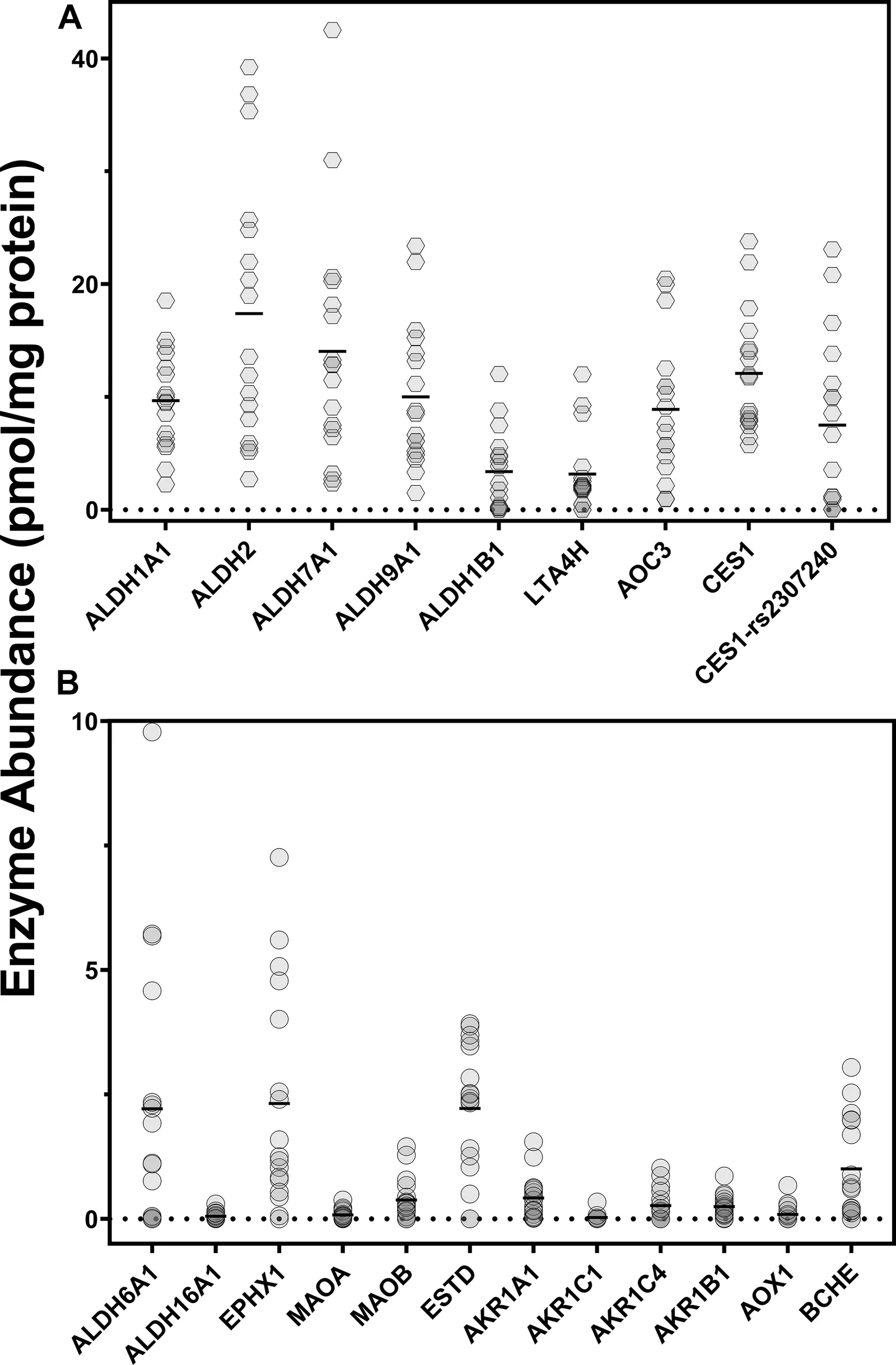
Total abundance of phase I (non-CYP) drug metabolizing enzymes in human skin. Scatter plots show combined protein abundances (pmol/mg protein) across both epidermal/dermal cytosol and membrane from 17 human skin samples. (A) Higher abundance enzymes, primarily from the aldehyde dehydrogenase family and related enzymes. (B) Lower abundance enzymes. Each circle represents an individual donor. Horizontal bars indicate the mean protein abundance for each enzyme.

The non-CYP enzymes quantified in 2 layers of human skin included members of the aldehyde dehydrogenase (ALDH) family—ALDH1A1, ALDH2, ALDH7A1, ALDH9A1, and ALDH1B1—alongside leukotriene A4 hydrolase (LTA4H), amine oxidase copper-containing 3 (AOC3), and carboxylesterase 1 (CES1) (Fig. 3A), including its splice variant ∗∗EST1-rs2307240. Quantitative proteomic analysis (*n* = 17) revealed a wide range of expression levels across these enzymes. Among the ALDH isoforms, ALDH2 displayed the highest mean abundance (17.4 pmol/mg protein), followed by ALDH7A1 (14.0 pmol/mg protein) with some individuals reaching levels exceeding 40 pmol/mg protein, then ALDH9A1 (10 pmol/mg protein), ALDH1A1 (9.67 pmol/mg protein), and AOC3 (8.90 pmol/mg protein). CES1 was also highly expressed (12.1 pmol/mg protein). In contrast, enzymes such as ALDH1B1 (3.37 pmol/mg protein) and LTA4H (3.16 pmol/mg protein) exhibited lower mean expression.

Interindividual variability was notable across the dataset. The highest %CV were observed for LTA4H (109.0%), CES1-rs2307240 (102%), and ALDH1B1 (106%), indicating substantial variability in expression levels among individuals. Conversely, EST1 (45%) and ALDH1A1 (45%) showed the least variability. This pattern is further supported by the 95% confidence intervals; for instance, the abundance of ALDH2 ranged from 11.3 to 23.4 pmol/mg protein, whereas ALDH1B1 ranged from 1.54 to 5.20 pmol/mg protein. The range of enzyme concentrations also varied widely, with ALDH2 and CES1 showing the broadest ranges (21.9 and 18.1 pmol/mg protein, respectively), whereas ALDH1B1 and LTA4H were more tightly distributed. These results highlight the heterogeneity of non-CYP enzyme expression in human skin and underscore the importance of considering both mean abundance and interindividual variability in metabolic profiling.

Several additional non-CYP and non-UDP-glucuronosyltransferase (non-UGT) enzymes with low expression levels were also quantified in human skin (*n* = 17) (Fig. 3B). These included members of the aldehyde dehydrogenase family (ALDH16A1 and ALDH6A1), epoxide hydrolase 1 (EPHX1), monoamine oxidases (MAOA, MAOB), esterases (ESTD and BCHE), aldo-keto reductases (AKR1A1, AKR1B1, AKR1C1, AKR1C4), and amine oxidase copper-containing 1 (AOX1). Among these, ESTD and EPHX1 displayed the highest mean abundances (2.22 and 1.00 pmol/mg protein, respectively), while enzymes such as ALDH16A1, and AKR1C1 were expressed at very low levels (<0.5 pmol/mg protein). Variability in expression was considerable across most enzymes, with %CV ranging from 59% for ESTD to 328% for AKR1C1, indicating substantial interindividual differences. Notably, ALDH16A1 and AOX1 all displayed %CVs exceeding 150%, whereas MAOB and MAOA showed slightly lower variability (115% and 130%, respectively). The relatively low mean and high variability of these enzymes underscore the need to account for potential interindividual differences in cutaneous metabolism, particularly for enzymes present at low but possibly functionally relevant levels.

### 3.12 Total expression and variability of phase II enzymes in dermis and epidermis of human skin in both membrane and cytosolic fractions

**Fig. 4:**
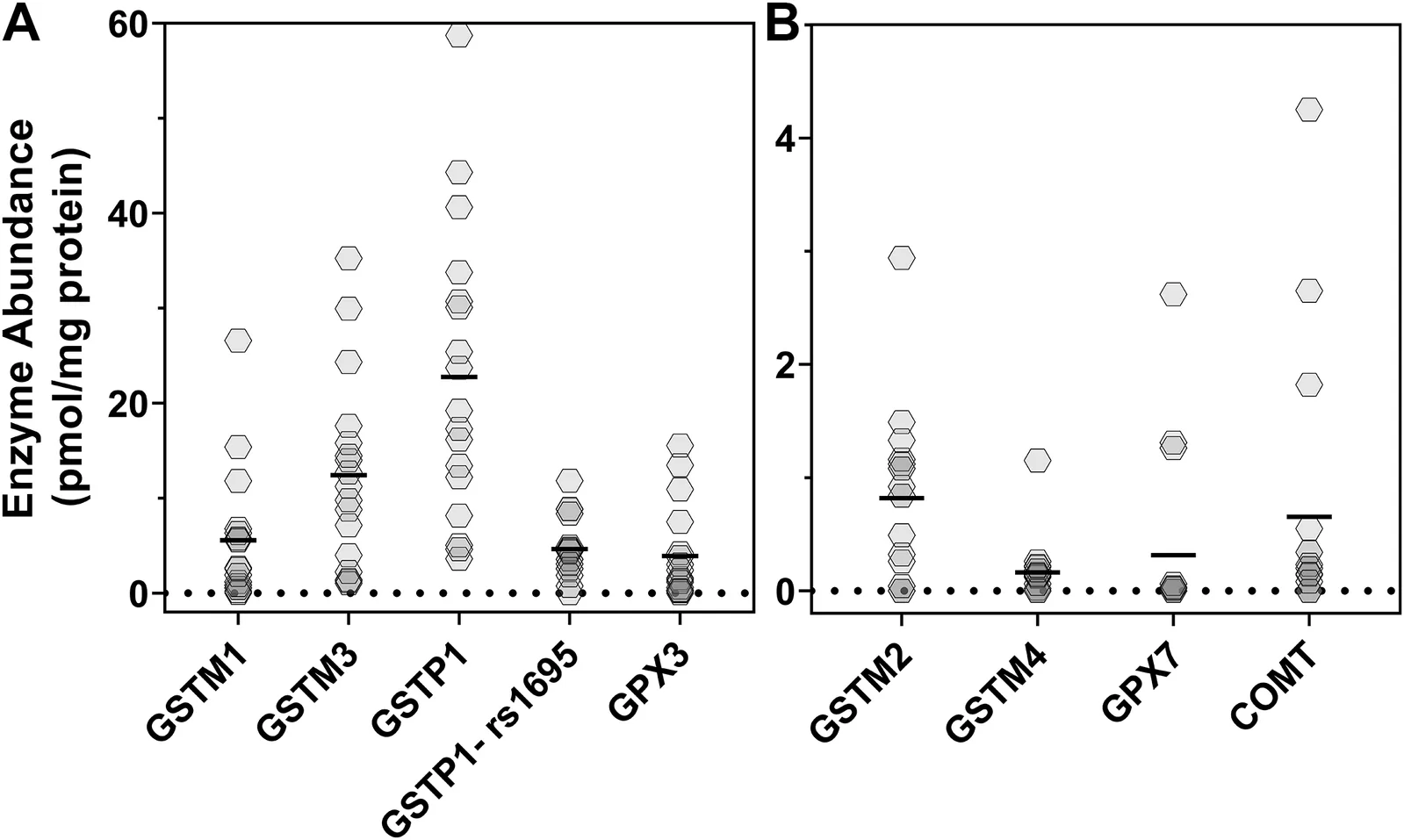
Total abundance of phase II metabolizing enzymes in human skin. Scatter plots show combined protein abundances (pmol/mg protein) from epidermal/dermal cytosol and membrane fractions across 17 human skin samples. (A) Higher abundance enzymes. (B) Lower abundance enzymes. Each symbol represents an individual donor. Horizontal bars indicate the mean protein abundance for each enzyme. GSTP1-rs1695 represents a known GSTP1 polymorphism.

Expression of phase II enzymes (Fig. 4, A and B) was measured in both skin layers. The data included glutathione S-transferases (GSTM1–4, GSTP1, and GSTP1-rs1695), glutathione peroxidases (GPX3, GPX7), and catechol-O-methyltransferase (COMT). Among the GST isoforms, GSTP1 showed the highest mean expression (22.8 ± 15.5), followed by GSTM3 (12.4 ± 10.1), whereas GSTM4 exhibited the lowest (0.16 ± 0.27). CV values highlighted the variability in expression, with GSTM4 (165%) and COMT (175%) showing particularly high variation, suggesting heterogeneous expression across individuals (Fig. 4B). Notably, GPX7 exhibited the highest CV (233%), despite its low mean value (0.31), indicating substantial interindividual variability in expression. The 95% CIs further reflected the spread of data, with GSTM3 (7.25–17.6) and GSTP1 (14.8–30.7) showing the broadest ranges (Fig. 4A). These findings suggest differential regulation and expression variability of these enzymes, which may be relevant in understanding individual differences in oxidative stress response and detoxification capacity.

### 3.13 Resolving enzyme diversity in skin cytosol: Polymorphisms, isoforms, and peptide-specific quantification

#### 3.13.1 Cytosolic metabolizing enzymes

Twenty-one enzymes were quantified in the epidermis cytosol and 29 in the dermis cytosol. There was a high degree of similarity between the 2 fractions (Supplemental Table 9). Table 3 summarizes the cytosolic enzymes quantified. There is evidence of 2 polymorphisms of GSTP1 in the dermis cytosol (Supplemental Table 10). The Uniprot consensus sequence accounts for most of the abundance and has the sequence YISLIYTNYEAGK at position 104 and is present in all samples. The minor component present in 13 of the 17 samples has valine replacing the isoleucine at position 105, so the unique peptide is YVSLIYTNYEAGK (Supplemental Table 10). These 2 peptides correspond to the canonical GSTP1 sequence and the well described rs1695 variant, respectively, and the presence of the valine-containing peptide in 13 of 17 donors indicates that both alleles are represented within the cohort. Thus, at least part of the interindividual variability in GSTP1 abundance is expected to reflect true genetic heterogeneity in GSTP1 sequence rather than technical variability alone. The situation for CES1, the abundant enzyme in liver is different. In several epidermal samples, the peptide FTPPQPAEPWNFVK is detected. This is not in the Uniprot consensus sequence, where FTPPQPAEPWSFVK is preferred. However, the serine-containing peptide is not found in the dermal samples so the protein is tentatively assigned as CES1 (74S→N) in all samples. In 2 dermal samples (C40 and C53) one ESTD peptide, AYDATHLVK, gave rise to intensities several orders of magnitude above those of the other peptides. The Hi3 quantification was therefore based on the second to fourth most intense peptides. Most of the dermal samples (C36, C37, C40, C44, C46, C49b, C50, C52, C53, C57, C62, and C69) also showed very high intensity for a single glutathione S-transferase Mu 3 (GSTM3) peptide (VDIIENQVMDFR) and again the Hi3 quantification was based on the second to fourth most intense peptides. This peptide was not seen in the epidermal samples. There is evidence of fatty acid dehydrogenase (ALDH3A2) and of CYP20A1 in most of the epidermal samples, but these proteins fall BLOQ. Bifunctional EPHX2 is found in some epidermal samples and also falls BLOQ. Similarly, GPX1 is detected but not quantified in dermal cytosol (Supplemental Table 9). Dermal aldo-keto reductase family 1 member C3 (AKR1C3) could equally well be assigned to aldo-keto reductase family 1 member C1 (AKR1C1) but the epidermal samples contain peptides unique for AKR1C3, so the dermal protein is tentatively assumed to be AKR1C3. The epidermal cytosolic samples fell into 2 very distinct groups.

**Table 3:** Drug metabolizing enzymes found in the epidermal and dermal cytosol of Caucasian human skin samples

Cytosolic Enzymes	Number of Peptides		Abundance (Epidermis Cytosol) Mean (pmol/mg) and CV (%)		Abundance (Dermis Cytosol) Mean (pmol/mg) and CV (%)	
	Epidermis	Dermis				
ALB1	11	8	2.63	114	0.72	95
ALDH1A1	32	31	3.7	97	4.62	48
ALDH1A2	5	5	0.21	148	0.09	154
ALDH2	37	33	9.71	109	5.01	50
ALDH3A1	21	23	1.67	95	1.06	77
ALDH4A1	10	3	0.19	177	0.01	178
ALDH7A1	36	37	7.67	114	6.27	55
ALDH9A1	28	31	3.68	102	3.66	55
ALDH16A1	0	2	0	—	0.01	321
AOC3	8	17	0.2	212	1.16	77
AOX1	0	20	0	—	0.09	195
AKR1A1	7	8	0.17	207	0.07	147
AKR7A2	0	3	0	—	0.01	254
AKR1B1	8	13	0.1	114	0.08	109
AKR1C2	7	7	0.02	229	0.01	254
AKR1C3 (AKR1C1)	6	8	0.02	148	0.03	328
CES1 (74S→N)	35	33	7.49	102	11.5	47
CHLE (cholinesterase)	5	7	0.23	163	0.77	111
EPHX2∗	0	15	0	—	0.23	160
ESTD	9	13	0.91	99	0.85	63
GSTM1	21	21	2.28	132	3.27	190
GSTM2	18	16	0.47	100	0.3	225
GSTM3	7	10	0.02	318	0.21	150
GSTP1	21	20	10.9	109	9.61	55
GSTP1 (105 I→V)	0	19	0	—	1.33	137
GGT5	0	5	0	—	0.02	173
GPX3	0	11	0	—	1.14	225
GPX7	0	2	0	—	0.31	233
LKHA4	35	34	1.91	149	1.2	85

—, not detected.

### 3.14 Distinct subgroups of skin samples defined by total protein abundance and molecular weight profiles

Samples C24, C36, C40, C43, C44, C46, C50, C53, and C69 gave a total of 15k–18k pmol/mg protein, corresponding to a weighted average molecular weight of 55–65 kDa. Samples C37, C47, C49a, C52, C54, C62, and C64, by contrast gave totals of only 7k–8k pmol/mg protein and weighted average molecular weights of 120–135 kDa; large proteins were therefore overrepresented. Only C49b, with 10k pmol/ mg protein and a weighted average molecular weight of 95 kDa fell between these extremes. The 7 samples in the second group had barely detectable abundances of enzymes. C49b behaved similarly to the first group, where the total enzyme abundance was 72–133 pmol/mg protein. The pattern in the dermis was quite different. Although samples C37, C43, and C62 had weighted average molecular weights of above 100 kDa, their total enzyme aligned well with the other samples. These ranged from 33 to 98 pmol/mg protein.

### 3.15 Correlation of cytosolic enzyme expression between epidermis and dermis

A strong positive correlation (Fig. 2B) in the abundance of cytosolic drug metabolizing enzymes between the epidermal and dermal layers of human skin was seen (Spearman’s *ρ* = 0.68, *P* < .0001). Each point represents an individual protein, with the dotted line indicating perfect agreement between layers. This result suggests a high degree of overall concordance in cytosolic enzyme expression across the 2 layers, despite some layer-specific variations.

#### 3.15.1 Membrane enzymes

Twenty-seven enzymes were quantified in the dermal membrane and 20 in the epidermal membrane (Supplemental Table 8 for individual samples). In addition, there is evidence of NADPH-CYP reductase in both epidermal and dermal membrane but it falls BLOQ. In general the membrane fractions were somewhat richer in enzymes and transporters than the cytosolic fractions (see Supplemental Material for full data). Table 4 shows the enzymes quantified in the membrane fractions. It is remarkable that GSTM3 is found predominantly in the membrane fractions. This is annotated to cytosol in the Uniprot database, albeit inferred from the evidence of a single study.

**Table 4:** Drug metabolizing enzymes found in the epidermal and dermal membrane fractions of Caucasian human skin

Membrane Enzymes	Number of Peptides		Abundance (Epidermis Membrane) Mean (pmol/mg) and CV (%)		Abundance (Dermis Membrane) Mean (pmol/mg) and CV (%)	
	Epidermis	Dermis				
ALDH1A1	12	15	0.56	93	0.78	79
ALDH2	13	17	1.68	86	0.98	84
ALDH3A1	2	3	0.03	235	0.01	223
ALDH3A2	10	9	1.16	113	0.56	96
ALDH4A1	0	2	0	—	1.80	107
ALDH6A1	0	4	0	—	2.21	124
ALDH7A1	2	2	0.06	176	0.02	229
ALDH9A1	10	8	1.67	148	1	97
ALDH16A1	2	0	0.05	177	0	—
ALDH1B1	0	3	0	—	0.02	135
AKR1A1	3	2	0.10	217	0.08	121
AKR1B1	0	3	0	—	0.06	153
AKR1C4	2	3	0.06	163	0.2	132
AOC3	12	16	0.98	94	6.55	91
CES1	6	5	0.43	123	0.19	121
COMT	4	5	0.41	235	0.24	175
EPHX1	9	13	39.6	56	22.6	98
ESTD	0	4	0	—	0.45	82
GPX3	4	5	1.89	162	0.89	153
GSTM1	4	0	0.01	239	0	—
GSTM2	2	4	0.03	260	0.02	193
GSTM3	9	10	4.30	186	7.9	82
GSTM4	0	4	0	—	0.16	165
GSTP1	0	9	0	—	2.22	100
GSTP1 (105 I→V)	9	9	3.16	68	0.17	202
LKHA4	0	2	0	—	0.05	149
MAOA	0	4	0	—	0.08	130
MAOB	4	7	0.14	211	0.23	109

—, not detected.

There is evidence of MGST3 in the dermal membrane fraction, but a single peptide, VLYAYGYYTGEPSK, contributes most of the intensity, with 2 other peptides present but BLOQ. Similarly, bifunctional EPHX2 falls BLOQ in the epidermis. The peptide SVINDPIYK is shared between UGT2B17 and UGTB15. It contributes most of the intensity for these proteins, although other peptides are detected. The data here indicate the presence of one or more proteins in the UGT2B series in the dermis but quantification will require further experimentation. CYP4F2 is also detected in the dermis but at levels below the lower limit of quantification.

### 3.16 Subcellular localization of key enzymes and transporters

Table 5 shows the locations of the enzymes quantified as annotated at www.Uniprot.org. Much of the skin metabolic activity is devoted to glutathione metabolism, but there are also abundant enzymes involved in redox metabolism and a surprisingly large amount of liver carboxyesterase 1. Table 6 shows the annotated location of transporters in the cell. Almost all the transporters quantified, especially the abundant ADT transporters are annotated to the mitochondrion inner membrane.

**Table 5:** Type of enzymes and their locations in human skin

Uniprot ID	Description	Annotated to	Principally Found (Abundance pmol/mg)	Also Found
Glutathione metabolism				
ESTD	S-formylglutathione hydrolase	Cytoplasm	EC 0.9	DC, DM
GSTP1	Glutathione S-transferase P	Cytoplasm, mitochondrion, nucleus	EC 10.9	DC, DM
GSTP1 (105 I→V)	Glutathione S-transferase P	Cytoplasm, mitochondrion, nucleus	EM 3.2	DM, DC
GSTM1	Glutathione S-transferase Mu 1	Cytoplasm	DC 3.2	EC, EM
GSTM2	Glutathione S-transferase Mu 2	Cytoplasm	EC 0.5	DC, EM, DM
GSTM3	Glutathione S-transferase Mu 3	Cytoplasm	DM 7.9	EM, EC, DC
GSTM4	Glutathione S-transferase Mu 4	Cytoplasm	DM 0.2	
GPX1	Glutathione peroxidase 1	Cytoplasm, mitochondrion	DM 0.01	DC
GPX3	Glutathione peroxidase 3	Secreted	EM 1.9	DC, DM
GPX7	Glutathione peroxidase 7	Secreted, endoplasmic reticulum	DC 0.3	
GGT5	Glutathione hydrolase 5	Membrane	DC 0.03	
Dehydrogenases				
ALD16A1	Aldehyde dehydrogenase family 16 member A1		EM 0.05	
AK1A1	Alcohol dehydrogenase [NADP(+)]	Cytoplasm, apical plasma membrane	EC 0.2	DC, EM, DM
AL1A1	Retinal dehydrogenase 1	Cytoplasm	DC 4.6	EC, DM, EM
AL1A2	Retinal dehydrogenase 2	Cytoplasm	EC 0.2	DC
AL1B1	Aldehyde dehydrogenase X mitochondrial	Mitochondrion, cytosol	EC 2.6	DC, DM
AL3A1	Aldehyde dehydrogenase, dimeric NADP-preferring	Cytoplasm	EC 1.7	DC, DM, EM
AL3A2	Fatty aldehyde dehydrogenase	Endoplasmic reticulum, cytosol	EM 1.2	EC, DM
AL4A1	Delta-1-pyrroline-5-carboxylate dehydrogenase	Mitochondrion, cytosol	DM 1.8	EC, DC
AL7A1	*α*-Aminoadipic semialdehyde dehydrogenase	Cytoplasm, mitochondrion, nucleus	EC 7.7	DC, EM, DM
AL9A1	4-Trimethylaminobutyraldehyde dehydrogenase	Cytoplasm	EC 3.7	DC, EM, DM
ALDH2	Aldehyde dehydrogenase, mitochondrial	Mitochondrion	EC 9.7	DC, DM, EM
MMSA	Methylmalonate-semialdehyde dehydrogenase [acylating], mitochondrial		DC 2.2	
Reductases				
ALDR	Aldose reductase	Cytoplasm	EC 0.1	DC, DM
AK1C1	Aldo-keto reductase family 1 member C1	Cytoplasm	DC 0.03	
AK1C2	Aldo-keto reductase family 1 member C2	Cytoplasm	EC 0.02	DC
AK1C3	Aldo-keto reductase family 1 member C3	Cytoplasm	EC 0.02	
AK1C4	Aldo-keto reductase family 1 member C4	Cytoplasm	DM 0.2	EM
ARK72	Aflatoxin B1 aldehyde reductase member 2	Mitochondrion, cytoplasm, Golgi apparatus	DC 0.01	
Oxidases				
AOC3	Membrane primary amine oxidase	Cell membrane	DC 6.6	EM, DC, EC
AOFB	Amine oxidase [flavin-containing] B	Mitochondrion outer membrane	DM 0.2	EM
AOFA	Amine oxidase [flavin-containing] A	Mitochondrion outer membrane	DM 0.08	
AOX1	Aldehyde oxidase	Cytoplasm	DC 0.09	
Hydrolases				
LKHA4	Leukotriene A-4 hydrolase	Cytoplasm	EC 1.9	DC, DM
HYEP	Epoxide hydrolase 1	Endoplasmic reticulum	DM 1.2	EM, DC
Esterases				
CES1 (74S→N)	Liver carboxylesterase 1		DC 11.5	EC, DM, EM
CHLE	Cholinesterase		DC 0.8	
Other				
COMT	Catechol O-methyltransferase		EM 0.4	DM

DC, dermis cytosol; DM, dermis membrane; EC, epidermis cytosol; EM, epidermis membrane.

**Table 6:** Transporter functions and locations

SLC Transporter	Location	Function and Drugs Transported
GTR1∗–GLUT1/SLC2A1	Cell membrane	Glucose transporter
B3AT–SLC4A1 (Band 3 anion transport protein)	Basolateral cell membrane	Mediates electroneutral anion exchange across the cell membrane
S12A2–SLC12A2	Basolateral cell membrane	Cation – chloride cotransporter
TXTP–SLC25A1	Mitochondrion inner membrane	Catalyzes exchange of citrate and malate
PTP–SLC25A3	Mitochondrion inner membrane	Transports phosphate or copper ions across the mitochondrial inner membrane
ADT1–SLC25A4 (ADP/ATP translocase 1)	Mitochondrion inner membrane	ADP:ATP antiporter
ADT2–SLC25A5 (ADP/ATP translocase 2)	Mitochondrion inner membrane	ADP:ATP antiporter
ADT3–SLC25A6 (ADP/ATP translocase 3)	Mitochondrion inner membrane	ADP:ATP antiporter
M2OM–SLC25A11	Mitochondrion inner membrane	Catalyzes exchange of 2-oxoglutarate and malate
S2512–SLC25A12	Mitochondrion inner membrane	Mitochondrial aspartate/glutamate antiporter
SCMC1–SLC25A24	Mitochondrion inner membrane	Mediates the transport of adenyl nucleotides through the inner mitochondrial membrane
S35F3–SLC35F3	Membrane	Mediates thiamine transport
CTL1–SLC44A1	Cell membrane, mitochondrion outer membrane	Choline/H+ antiporter
CTL2–SLC44A2	Cell membrane, mitochondrion outer membrane	Choline/H+ antiporter

## 4 Discussion

The skin, as the body’s largest organ, serves as both a physical and biochemical barrier, regulating exposure to environmental agents and topically applied drugs. Despite its clinical and pharmacological significance, there is, to date, no detailed protein level understanding of drug metabolizing enzymes and transporters in the skin. Our study represents the first comprehensive proteomic analysis of metabolizing enzymes and transporters across different skin layers and subcellular fractions, characterizing over 1000 proteins in total and providing novel insights into their spatial distribution and interindividual variability. Our findings are consistent with prior global skin proteomic studies that analyzed whole-tissue homogenates or single layers and reported abundant structural, immune-related and xenobiotic-metabolizing proteins in human skin.^11^^,^^50^^,^^51^ The present work extends these datasets by providing proteomic quantitative profiling across both dermis and epidermis, each resolved into cytosolic and membrane fractions. The skin samples presented significant technical challenges because of their high fat content and the presence of insoluble material, resulting in weaker membrane samples with total intensities typically around 10^9^ compared with 10^10^ in cytosolic samples. Additionally, the total number of peptides detected was below 10,000, considerably lower than the 20,000 to 30,000 typically observed in liver microsome samples using identical instrumentation. In addition, we found that external standards used for quantification failed to mix completely with the analytes. These challenges necessitated the adaptation of the HiN quantification approach, so as to avoid the need for external standards. By calculating a weighted average molecular weight for each sample, we were able to use the total sample, rather than an external standard, for normalization of abundances (the HiNt method, to be reported in full elsewhere). In addition, our inclusion of nonunique peptides with proportional distribution of intensity among matched proteins expanded our coverage beyond what would be possible with unique peptides alone, allowing the quantification of similar proteins, including polymorphisms. Many anomalously abundant peptides originated from highly abundant structural, extracellular, or immune-related proteins (eg, collagens, keratins, and immunoglobulins) or from short, poorly annotated fragments, where full-length protein expression would be expected to yield multiple peptides. Dominance of single peptides therefore most likely reflects structural protein abundance, proteolytic resistance, differential ionization efficiency, or accumulation of stable cleavage products rather than true high-level expression. Detection of trypsin-derived peptides and low level unlabeled peptide from QconCAT standards further indicates unavoidable analytical artifacts and reagent carry-over in fibrous tissues such as skin. These peptides were explicitly controlled for during data processing and excluded from HiN quantification where appropriate. Use of a modified HiN normalization strategy minimized bias from dominant peptides, ensuring robust enzyme and transporter quantification. A striking finding of our study was the limited detection of typical membrane-bound drug metabolizing enzymes, particularly CYP enzymes, in both epidermal and dermal layers. For instance, only 2 CYP enzymes, CYP1A2 and CYP20A1 were detected across all cytosolic samples, and both were present in very low abundance, consistent with previous report suggesting that CYP-mediated metabolism is negligible in human skin.^11^ This observation aligns with previous studies showing that CYP expression in skin is markedly lower than in liver, with skin metabolism estimated to be only 10%–30% of hepatic activity.^52^, ^53^, ^54^ The CYP450 isoforms typically involved in xenobiotic metabolism (CYP1A, CYP2C, CYP2D, and CYP3A) were mostly absent or present at levels below quantification limits in the skin samples.^55^ This limited expression of membrane-bound metabolizing enzymes suggests that the skin's role in drug biotransformation differs substantially from that of the liver, with implications for topical drug delivery and safety assessment. The absence of significant CYP activity is consistent with the absence of significant first-pass metabolism and the retention of activity of many topically applied drugs.^55^ In contrast to membrane-bound enzymes, cytosolic drug metabolizing enzymes were readily quantifiable and revealed a metabolic profile distinct from liver tissue.^56^ GSTs and ALDHs were especially abundant, with GSTP1 showing the highest mean expression (22.8 pmol/mg protein) followed by GSTM1 at 12.4 pmol/mg protein in both skin layers. The high expression of GSTP1 and GSTM1, highlights the skin's capacity for conjugation and detoxification reactions, which may serve as a biochemical barrier against xenobiotics and environmental toxins. This finding is consistent with the skin’s function as a first-line defense against environmental insults.^57^^,^^58^ Although GSTM3 is typically cytosolic, it has been shown to highly expressed in the membrane (4.30 pmol/mg protein in epidermis membrane; 7.9 pmol/mg protein in dermis membrane). This dual localization supports its potential role in maintaining redox balance in the skin, which is particularly relevant in an environment chronically exposed to UV light, pollution, and mechanical damage.

Among ALDHs, ALDH2 displayed the highest abundance (17.4 pmol/mg protein) in all layers, indicating significant capacity for aldehyde detoxification and potentially retinoic acid metabolism in skin.^59^ Interestingly, ALDH1A1 a key enzyme in retinoid metabolism,^60^^,^^61^ was consistently expressed across all 4 skin fractions with a dermis cytosol concentration of 4.6 pmol/mg protein. Although classically associated with neural tissues, its presence in all fractions may reflect roles in local retinoic acid synthesis and as promising target for pharmacological manipulation of melanin pigmentation.^62^ Its expression may influence therapeutic response in conditions such as acne, psoriasis, and aging.

High expression of CES1 emerged as another noteworthy finding, with expression levels reaching up to 19.6 pmol/mg protein in both dermis and epidermis, although these values remain ∼18-fold lower than hepatic concentrations (∼360 pmol/mg protein).^12^^,^^26^ This challenges the traditional view of CES1 as primarily a liver enzyme and supports emerging evidence of its broader tissue distribution.^63^ The detection of both CES1 and its splice variant suggests polymorphic expression that could contribute to interindividual variability in cutaneous drug metabolism.^64^

The high interindividual variability we observed in several enzyme families should be interpreted in the context of functional redundancy. For many oxidoreductases (eg, multiple ALDHs) and transferases (eg, GSTP1 and GSTM1/3), multiple isoforms were coexpressed across skin layers, suggesting that reduced expression or activity of a single isoform due to genetic polymorphisms may be partially buffered by overlapping catalytic capacities within the same family. In addition to quantitative variability, GSTP1 exhibited qualitative diversity at the peptide level, with detection of both the consensus peptide YISLIYTNYEAGK and the variant peptide YVSLIYTNYEAGK (GSTP1-rs1695) in dermal cytosol. The rs1695 polymorphism is known to alter amino acid sequence and has been associated with differences in GSTP1 activity in other tissues, so its detection here suggests that genetic variation may contribute to interdonor differences in cutaneous GSTP1 function.^65^, ^66^, ^67^, ^68^ This supports the view that, for some enzymes, observed variability in protein abundance reflects a combination of analytical, environmental, and inherited genetic factors.^69^^,^^70^

In contrast, CES1 quantified here at relatively high levels but with fewer equally abundant hydrolase alternatives showing comparable substrate specificity, may have lower functional redundancy^63^; substantial loss of CES1 activity could therefore exert greater influence on cutaneous disposition of esterified drugs. Despite structural and functional differences between dermis and epidermis, the study revealed strong positive correlations in protein expression between layers (Spearman’s *ρ* = 0.85 for transporters, *ρ* = 0.68 for cytosolic enzymes; Fig. 2, A and B). Although the majority of quantified proteins followed the expected concordance between epidermal and dermal layers, a small subset consistently deviated from the line of unity. These proteins showed marked layer-enriched expression and were therefore highlighted in Fig. 2, A and B. Such layer-skewed proteins may be of particular interest as potential indicators of successful epidermal–dermal separation and could serve as pragmatic quality control markers in future skin proteomic studies. Although the present study was not designed to formally validate dissection markers, the reproducible deviation observed across donors suggests that these proteins may warrant further evaluation. This finding suggests conserved metabolic and transport capacity across skin compartments while maintaining some layer-specific variations.^71^ In light of these conserved yet subtly layer-specific patterns, our quantitative data enable inferences regarding local dermal clearance, cutaneous first-pass metabolism, and transporter mediated disposition. Quantitatively, the enzyme profile indicates that local dermal clearance is driven predominantly by non-CYP pathways, particularly ALDHs, CES1, and glutathione-dependent conjugation, rather than classical hepatic CYP isoforms.^11^^,^^21^^,^^52^, ^53^, ^54^ High expression of ALDHs (eg, ALDH2 and ALDH1A1) and GSTs (notably GSTP1 and GSTM3), together with measurable epoxide hydrolase and other oxidoreductases, suggests substantial capacity for detoxification of reactive aldehydes, electrophiles, and pro-oxidant species generated from topically applied drugs or via UV and pollution-induced lipid peroxidation.^21^^,^^56^, ^57^, ^58^ For esterified or soft-drug structures, the relatively high abundance of CES1 in both epidermal and dermal fractions supports efficient local hydrolysis, consistent with cutaneous metabolism of ester-containing drugs such as diclofenac and methyl salicylate, and implies that skin may act as a quantitatively relevant clearance organ at the site of administration.^17^^,^^21^^,^^72^ Taken together, the low abundance of CYP enzymes, the predominance of phase II conjugation pathways, and comparable enzyme profiles between epidermis and dermis indicate that cutaneous first-pass effects are governed mainly by rapid conjugation and hydrolysis in viable epidermis and upper dermis, rather than oxidative CYP-mediated turnover.^11^^,^^21^^,^^52^, ^53^, ^54^, ^55^ For topically applied drugs with high intrinsic clearance via GSTs or CES1, a substantial fraction of the dose may therefore be metabolized within skin before systemic entry, attenuating systemic exposure while shaping local parent and metabolite concentrations.^9^^,^^16^^,^^21^^,^^23^ These quantitative abundance values can be incorporated as Vmax-scaling inputs in dermal PBPK models to distinguish compounds undergoing local metabolic trapping from those more likely to escape into the systemic circulation.^32^

With respect to transporter mediated disposition, the dominance of mitochondrial SLC transporters (eg, SLC25A4/5/6) suggests tight coupling between energy metabolism and xenobiotic handling in skin, whereas sparse detection of nonmitochondrial SLCs and the absence of quantifiable ABC transporters imply a limited role for active efflux or vectorial transport across the dermal–epidermal interface under basal conditions.^11^^,^^12^^,^^19^^,^^72^^,^^73^ This supports dermal pharmacokinetic scenarios in which transdermal permeation is largely permeability-driven, with transporters primarily influencing intracellular compartmentation, mitochondrial exposure, and local toxicity rather than net flux.^22^^,^^24^^,^^32^^,^^72^^,^^74^ Transporters, although less abundant than enzymes, revealed interesting trends. Several mitochondrial SLC family members, such as SLC25A5 and SLC25A3 were highly expressed and consistently quantified. These function as ADP/ATP translocases and phosphate/copper ions transport in the mitochondrial inner membrane.^74^ This finding suggests that energy metabolism and mitochondrial function are critical aspects of skin physiology, reflecting the significant energy requirements of keratinocyte differentiation and barrier function maintenance.^73^ The limited detection of nonmitochondrial drug transporters raises important questions about the predominant mechanisms of drug transport across the skin. Although some nonmitochondrial SLC transporters (S12A2/SLC12A2, CTL1/SLC44A1, and CTL2/SLC44A2) were identified, their expression levels were generally low compared with mitochondrial transporters. The quantitative protein abundance data generated in this study provide critical missing parameters for dermal PBPK models, addressing a key bottleneck in topical drug development.^75^ These findings can directly enhance the predictive performance of dermal PBPK models for topical drug delivery. Such models can inform decision-making across drug product development and lifecycle management for both innovator and generic products and support international initiatives to reduce animal testing by enabling validated nonclinical methods for predicting human skin exposure. Enzymes like CES1, GSTP1, and ALDH1A1, together with mitochondrial transporters, can now be incorporated with greater accuracy. The high donor-to-donor variability, with coefficients of variation exceeding 100% for many proteins, observed further emphasizes the importance of modeling not just mean abundance, but population-level distributions. Donors in this study were exclusively Caucasian and almost all female (16 women, 1 man) aged 24–69 years, all of Caucasian ethnicity. This demographic limitation may affect generalizability to other populations and highlights the need for more diverse sample sets in future investigations. In addition, this study focused exclusively on healthy skin. Disease states such as psoriasis, atopic dermatitis are associated with alterations in barrier integrity, enzyme expression, and inflammatory pathways. Including diseased skin samples in future investigations would therefore be highly valuable to capture the full spectrum of variability relevant for topical drug disposition and dermal PBPK modeling. The present cohort was restricted to abdominoplasty tissue from predominantly female Caucasian donors, which has important implications for generalizability. Skin thickness is known to vary substantially across anatomical sites, typically ranging from approximately 0.5–4 mm, and is accompanied by site-specific differences in appendage density, vascularization, and exposure history.^76^ These structural and physiological variations are likely to influence the local expression of metabolizing enzymes and transporters. In addition, sex-, age-, and ethnicity-related differences in skin structure, barrier function, immune tone, and systemic hormonal milieu are also recognized determinants of cutaneous protein expression. Therefore, the abundances reported here should not be assumed to be directly transferable to male, non-Caucasian, pediatric, or elderly populations. Consequently, the current dataset is best viewed as a well characterized reference panel for healthy adult Caucasian abdominal skin, providing a foundation for future studies that extend to other anatomical sites, incorporate more balanced sex distributions, and include ethnically diverse cohorts, ultimately enabling more robust extrapolation of dermal PBPK models across populations. Although the study provides comprehensive protein quantification, functional validation of enzyme activities and their metabolic contributions remains desirable^72^; proteomic measurements do not distinguish between active and inactive protein, and this may be important in samples, such as these, containing high concentrations of trypsin. However, this research fundamentally challenges traditional views of skin as a metabolically inert barrier. The documentation of substantial enzymatic diversity, particularly in phase II metabolism, establishes skin as an active metabolic organ with distinct capabilities from liver tissue. The findings have immediate applications for both cosmetic and pharmaceutical product development. Understanding the skin’s metabolic and transport profile enables better prediction of product safety, efficacy, and potential for adverse reactions. The data support the development of improved topical formulations and delivery systems.

## Conflict of interest

Amin Rostami-Hodjegan is employee of Certara. All other authors declare no conflicts of interest.

## References

[1] Al-Majdoub Z.M., Cheong J., Mizuno K. (2025). Transporter expressions as part of required scaling factor to support in vitro in vivo extrapolation for blood-brain barrier drug permeability. Eur J Pharm Sci.

[2] Research and Markets (2024).

[3] Raina N., Rani R., Thakur V.K., Gupta M. (2023). New insights in topical drug delivery for skin disorders: from a nanotechnological perspective. ACS Omega.

[4] Buhse L., Kolinski R., Westenberger B. (2005). Topical drug classification. Int J Pharm.

[5] Hmingthansanga V., Singh N., Banerjee S., Manickam S., Velayutham R., Natesan S. (2022). Improved topical drug delivery: role of permeation enhancers and advanced approaches. Pharmaceutics.

[6] McGrath J.A., Uitto J., Barker J., Griffiths C., Bleiker T., Simpson R., Hussain W. (2024). Rook’s Textbook of Dermatology.

[7] Romani N., Holzmann S., Tripp C.H., Koch F., Stoitzner P. (2003). Langerhans cells – dendritic cells of the epidermis. APMIS.

[8] Wiegand C., Hewitt N.J., Merk H.F., Reisinger K. (2014). Dermal xenobiotic metabolism: a comparison between native human skin, four in vitro skin test systems and a liver system. Skin Pharmacol Physiol.

[9] Manevski N., Swart P., Balavenkatraman K.K. (2015). Phase II metabolism in human skin: skin explants show full coverage for glucuronidation, sulfation, N-acetylation, catechol methylation, and glutathione conjugation. Drug Metab Dispos.

[10] Smith S.A., Colley H.E., Sharma P. (2018). Expression and enzyme activity of cytochrome P450 enzymes CYP3A4 and CYP3A5 in human skin and tissue-engineered skin equivalents. Exp Dermatol.

[11] Couto N., Newton J.R.A., Russo C. (2021). Label-free quantitative proteomics and substrate-based mass spectrometry imaging of xenobiotic metabolizing enzymes in ex vivo human skin and a human living skin equivalent model. Drug Metab Dispos.

[12] Al-Majdoub Z.M., Achour B., Couto N. (2020). Mass spectrometry-based abundance atlas of ABC transporters in human liver, gut, kidney, brain and skin. FEBS Lett.

[13] Al-Majdoub Z.M., Couto N., Achour B. (2021). Quantification of proteins involved in intestinal epithelial handling of xenobiotics. Clin Pharmacol Ther.

[14] Eilstein J., Lereaux G., Arbey E., Daronnat E., Wilkinson S., Duche D. (2015). Xenobiotic metabolizing enzymes in human skin and SkinEthic reconstructed human skin models. Exp Dermatol.

[15] Sharma A.M., Novalen M., Tanino T., Uetrecht J.P. (2013). 12-OH-nevirapine sulfate, formed in the skin, is responsible for nevirapine-induced skin rash. Chem Res Toxicol.

[16] Oesch F., Fabian E., Oesch-Bartlomowicz B., Werner C., Landsiedel R. (2007). Drug-metabolizing enzymes in the skin of man, rat, and pig. Drug Metab Rev.

[17] Hagen M., Baker M. (2017). Skin penetration and tissue permeation after topical administration of diclofenac. Curr Med Res Opin.

[18] Olsson Gisleskog P.O., Perez Ruixo J.J., Westin A., Hansson A.C., Soons P.A. (2021). Nicotine population pharmacokinetics in healthy smokers after intravenous, oral, buccal and transdermal administration. Clin Pharmacokinet.

[19] Osman-Ponchet H., Gaborit A., Linget J.M., Wilson C.E. (2017). Expression of drug transporters in the human skin: comparison in different species and models and its implication for drug development. ADMET & DMPK.

[20] Luu-The V., Duche D., Ferraris C., Meunier J.R., Leclaire J., Labrie F. (2009). Expression profiles of phases 1 and 2 metabolizing enzymes in human skin and the reconstructed skin models Episkin and full thickness model from Episkin. J Steroid Biochem Mol Biol.

[21] Zhang Q., Grice J.E., Wang G., Roberts M.S. (2009). Cutaneous metabolism in transdermal drug delivery. Curr Drug Metab.

[22] Roberts M.S., Cheruvu H.S., Mangion S.E. (2021). Topical drug delivery: history, percutaneous absorption, and product development. Adv Drug Deliv Rev.

[23] Svensson C.K. (2009). Biotransformation of drugs in human skin. Drug Metab Dispos.

[24] Baron J.M., Holler D., Schiffer R. (2001). Expression of multiple cytochrome p450 enzymes and multidrug resistance-associated transport proteins in human skin keratinocytes. J Invest Dermatol.

[25] Smith G., Wolf C.R., Deeni Y.Y. (2003). Cutaneous expression of cytochrome P450 CYP2S1: individuality in regulation by therapeutic agents for psoriasis and other skin diseases. Lancet.

[26] Couto N., Al-Majdoub Z.M., Achour B., Wright P.C., Rostami-Hodjegan A., Barber J. (2019). Quantification of proteins involved in drug metabolism and disposition in the human liver using label-free global proteomics. Mol Pharm.

[27] El-Khateeb E., Achour B., Al-Majdoub Z.M., Barber J., Rostami-Hodjegan A. (2021). Non-uniformity of changes in drug-metabolizing enzymes and transporters in liver cirrhosis: implications for drug dosage adjustment. Mol Pharm.

[28] Eckersley A., Mellody K.T., Pilkington S. (2018). Structural and compositional diversity of fibrillin microfibrils in human tissues. J Biol Chem.

[29] Couto N., Malys N., Gaskell S.J., Barber J. (2013). Partition and turnover of glutathione reductase from *Saccharomyces cerevisiae*: a proteomic approach. J Proteome Res.

[30] Hu J., Van den Steen P.E., Sang Q.X., Opdenakker G. (2007). Matrix metalloproteinase inhibitors as therapy for inflammatory and vascular diseases. Nat Rev Drug Discov.

[31] Al-Majdoub Z.M., Al Feteisi H., Achour B. (2019). Proteomic quantification of human blood-brain barrier SLC and ABC transporters in healthy individuals and dementia patients. Mol Pharm.

[32] Tsakalozou E., Alam K., Babiskin A., Zhao L. (2022). Physiologically-based pharmacokinetic modeling to support determination of bioequivalence for dermatological drug products: scientific and regulatory considerations. Clin Pharmacol Ther.

[33] Food and Drug Administration (FDA) (2025).

[34] US Food and Drug Administration Science Board (2024).

[35] El-Khateeb E., Vasilogianni A.M., Alrubia S. (2019). Quantitative mass spectrometry-based proteomics in the era of model-informed drug development: applications in translational pharmacology and recommendations for best practice. Pharmacol Ther.

[36] Al-Majdoub Z.M., Freriksen J.J.M., Colbers A. (2025). Absolute membrane protein abundance of P-glycoprotein, breast cancer resistance protein, and multidrug resistance proteins in term human placenta tissue and commonly used cell systems: application in physiologically based pharmacokinetic modeling of placental drug disposition. Drug Metab Dispos.

[37] Cichon M.A., Elbe-Burger A. (2023). Epidermal/dermal separation techniques and analysis of cell populations in human skin sheets. J Invest Dermatol.

[38] Howard M., Achour B., Al-Majdoub Z., Rostami-Hodjegan A., Barber J. (2018). GASP and FASP are complementary for LC-MS/MS proteomic analysis of drug-metabolizing enzymes and transporters in pig liver. Proteomics.

[39] Vasilogianni A.M., El-Khateeb E., Achour B. (2022). A family of QconCATs (Quantification conCATemers) for the quantification of human pharmacological target proteins. J Proteomics.

[40] Al-Majdoub Z.M., Carroll K.M., Gaskell S.J., Barber J. (2014). Quantification of the proteins of the bacterial ribosome using QconCAT technology. J Proteome Res.

[41] Barber J., Al-Majdoub Z.M., Couto N. (2022). Label-free but still constrained: assessment of global proteomic strategies for the quantification of hepatic enzymes and transporters. Drug Metab Dispos.

[42] Vasilogianni A.M., Al-Majdoub Z.M., Achour B., Peters S.A., Rostami-Hodjegan A., Barber J. (2023). Proteomic quantification of receptor tyrosine kinases involved in the development and progression of colorectal cancer liver metastasis. Front Oncol.

[43] El-Khateeb E., Al-Majdoub Z.M., Rostami-Hodjegan A., Barber J., Achour B. (2021). Proteomic quantification of changes in abundance of drug-metabolizing enzymes and drug transporters in human liver cirrhosis: different methods, similar outcomes. Drug Metab Dispos.

[44] Koop B.F., Rowen L., Wang K. (1994). The human T-cell receptor TCRAC/TCRDC (C alpha/C delta) region: organization, sequence, and evolution of 97.6 kb of DNA. Genomics.

[45] Brignac-Huber L., Reed J.R., Backes W.L. (2011). Organization of NADPH-cytochrome P450 reductase and CYP1A2 in the endoplasmic reticulum–microdomain localization affects monooxygenase function. Mol Pharmacol.

[46] Alrubia S., Al-Majdoub Z.M., Achour B., Rostami-Hodjegan A., Barber J. (2022). Quantitative assessment of the impact of Crohn’s disease on protein abundance of human intestinal drug-metabolising enzymes and transporters. J Pharm Sci.

[47] Gautron A., Migault M., Bachelot L., Corre S., Galibert M.D., Gilot D. (2021). Human TYRP1: two functions for a single gene?. Pigment Cell Melanoma Res.

[48] Journe F., Id Boufker H., Van Kempen L. (2011). TYRP1 mRNA expression in melanoma metastases correlates with clinical outcome. Br J Cancer.

[49] Payne J.A., Xu J.C., Haas M., Lytle C.Y., Ward D., Forbush B. (1995). Primary structure, functional expression, and chromosomal localization of the bumetanide-sensitive Na-K-Cl cotransporter in human colon. J Biol Chem.

[50] Mikesh L.M., Aramadhaka L.R., Moskaluk C., Zigrino P., Mauch C., Fox J.W. (2013). Proteomic anatomy of human skin. J Proteomics.

[51] Dyring-Andersen B., Løvendorf M.B., Coscia F. (2020). Spatially and cell-type resolved quantitative proteomic atlas of healthy human skin. Nat Commun.

[52] Oesch F., Fabian E., Landsiedel R. (2018). Xenobiotica-metabolizing enzymes in the skin of rat, mouse, pig, guinea pig, man, and in human skin models. Arch Toxicol.

[53] Kazem S., Linssen E.C., Gibbs S. (2019). Skin metabolism phase I and phase II enzymes in native and reconstructed human skin: a short review. Drug Discov Today.

[54] Eilstein J., Léreaux G., Budimir N., Hussler G., Wilkinson S., Duché D. (2014). Comparison of xenobiotic metabolizing enzyme activities in ex vivo human skin and reconstructed human skin models from SkinEthic. Arch Toxicol.

[55] Chen Q., Wang T., Wu X., Yuan H., Wei Y., Xiao Y. (2024). The role of the cytochrome P450 superfamily in the skin. Expert Rev Mol Med.

[56] van Eijl S., Zhu Z., Cupitt J. (2012). Elucidation of xenobiotic metabolism pathways in human skin and human skin models by proteomic profiling. PLoS One.

[57] Tew K.D., Townsend D.M. (2012). Glutathione-s-transferases as determinants of cell survival and death. Antioxid Redox Signal.

[58] Karadag A.S., Uzuncakmak T.K., Ozkanli S. (2017). An investigation of cytochrome p450 (CYP) and glutathione S-transferase (GST) isoenzyme protein expression and related interactions with phototherapy in patients with psoriasis vulgaris. Int J Dermatol.

[59] Kedishvili N.Y. (2013). Enzymology of retinoic acid biosynthesis and degradation. J Lipid Res.

[60] Roos T.C., Jugert F.K., Merk H.F., Bickers D.R. (1998). Retinoid metabolism in the skin. Pharmacol Rev.

[61] Molotkov A., Duester G. (2003). Genetic evidence that retinaldehyde dehydrogenase Raldh1 (Aldh1a1) functions downstream of alcohol dehydrogenase Adh1 in metabolism of retinol to retinoic acid. J Biol Chem.

[62] Kleszczynski K., Slominski A.T. (2013). Targeting ALDH1A1 to treat pigmentary disorders. Exp Dermatol.

[63] Yerrakula G., Abraham S., John S. (2024). Major implications of single nucleotide polymorphisms in human carboxylesterase 1 on substrate bioavailability. Biotechnol Genet Eng Rev.

[64] Her L., Shi J., Wang X. (2023). Identification of regulatory variants of carboxylesterase 1 (CES1): a proof-of-concept study for the application of the Allele-Specific Protein Expression (ASPE) assay in identifying cis-acting regulatory genetic polymorphisms. Proteomics.

[65] Dasgupta R.K., Adamson P.J., Davies F.E. (2003). Polymorphic variation in GSTP1 modulates outcome following therapy for multiple myeloma. Blood.

[66] Saadat M. (2017). Evaluation of glutathione S-transferase P1 (GSTP1) Ile105Val polymorphism and susceptibility to type 2 diabetes mellitus, a meta-analysis. EXCLI J.

[67] Hu X., Xia H., Srivastava S.K. (1997). Activity of four allelic forms of glutathione S-transferase hGSTP1-1 for diol epoxides of polycyclic aromatic hydrocarbons. Biochem Biophys Res Commun.

[68] Sundberg K., Johansson A.S., Stenberg G. (1998). Differences in the catalytic efficiencies of allelic variants of glutathione transferase P1-1 towards carcinogenic diol epoxides of polycyclic aromatic hydrocarbons. Carcinogenesis.

[69] Vesell E.S. (1991). Genetic and environmental factors causing variation in drug response. Mutat Res.

[70] Chen L., Zhernakova D.V., Kurilshikov A. (2022). Influence of the microbiome, diet and genetics on inter-individual variation in the human plasma metabolome. Nat Med.

[71] Luszczynski K., Soszynska M., Komorowski M. (2024). Markers of dermal fibroblast subpopulations for viable cell isolation via cell sorting: a comprehensive review. Cells.

[72] Telaprolu K.C., Grice J.E., Mohammed Y.H., Roberts M.S. (2023). Human skin drug metabolism: relationships between methyl salicylate metabolism and esterase activities in IVPT skin membranes. Metabolites.

[73] Gutiérrez-Aguilar M., Baines C.P. (2013). Physiological and pathological roles of mitochondrial SLC25 carriers. Biochem J.

[74] Ruprecht J.J., Kunji E.R.S. (2020). The SLC25 mitochondrial carrier family: structure and mechanism. Trends Biochem Sci.

[75] Patel N., Clarke J.F., Salem F. (2022). Multi-phase multi-layer mechanistic dermal absorption (MPML MechDermA) model to predict local and systemic exposure of drug products applied on skin. CPT Pharmacometrics Syst Pharmacol.

[76] Sandby-Møller J., Poulsen T., Wulf H.C. (2003). Epidermal thickness at different body sites: relationship to age, gender, pigmentation, blood content, skin type and smoking habits. Acta Derm Venereol.

