# Supplementary material for: Proteomic profiling of metabolizing enzymes and transporters in 2 layers of Caucasian human skin
Source: Drug Metab Dispos. 2026 Apr 29;54(6):100310. doi: 10.1016/j.dmd.2026.100310 (PMC13494176; doi:10.1016/j.dmd.2026.100310)
Supplement: Supplementary file 1 [file mmc1.docx]

**Supplementary Information**

**Proteomic Profiling of Metabolising Enzymes and Transporters in Two Layers of Caucasian Human Skin**

Seun Seriki, Amin Rostami-Hodjegan, Stacey Warwood, Eleftheria Tsakalozou, Mingliang Tan, Jill Barber, Zubida M. Al-Majdoub

*Correspondence to: Dr. Z Al-Majdoub;

email: zubida.al- majdoub@manchester.ac.uk

**Supporting Information Contents**

**Supporting Results**

Supplemental Table 1: Demographic information of the skin donors

Supplemental Table 2: Total intensity due to all peptides and number of distinct peptide identities recorded for abdominoplasty skin samples from 17 Caucasian donors

Supplemental Table 3: Human peptides recorded at unexpectedly high intensity in one or more fraction of skin

Supplemental Table 4: The total intensity of unlabelled peptide as a percentage of the labelled peptide detected

Supplemental Table 5: Protein abundance of four fractions (17 samples each) of Caucasian human skin. Sample numbers correspond to donor age

Supplemental Table 6: SLC transporter peptides found in cytosolic fractions of Caucasian human skin

Supplemental Table 7: Protein abundance of transporters in dermis and epidermis membrane fractions from 17 human donors

Supplemental Table 8: Protein abundance of enzymes in dermis and epidermis membrane fractions from 17 human donors

Supplemental Table 9: Protein abundance enzymes in dermis and epidermis cytosol fractions from 17 human donors

Supplemental Table 10: The presence of GSTP1 and GSTP1(105 I→V) unique peptides YISLIYTNYEAGK and YVSLIYTNYEAGK respectively in four fractions of Caucasian human skin

| **Supplemental Table 1**: Demographic information of the skin donors | | | | |
| --- | --- | --- | --- | --- |
| Donor ID | Sample ID | Age | Sex | Ethnicity |
| 6177 | C46 | 46 | F | Cauc |
| 8557 | C47 | 47 | F | Cauc |
| 8564 | C37 | 37 | F | Cauc |
| 8409 | C44 | 44 | F | Cauc |
| 8566 | C64 | 64 | F | Cauc |
| 9045 | C24 | 24 | F | Cauc |
| 9042 | C54 | 54 | F | Cauc |
| 9024 | C36 | 36 | F | Cauc |
| 9031 | C52 | 52 | F | Cauc |
| 9385 | C69 | 69 | F | Cauc |
| 9399 | C50 | 50 | F | Cauc |
| 9802 | C49a | 49 | F | Cauc |
| 9821 | C62 | 62 | F | Cauc |
| 10272 | C49b | 49 | F | Cauc |
| 10280 | C53 | 53 | F | Cauc |
| 10861 | C40 | 40 | F | Cauc |
| 5403 | C43 | 43 | M | Cauc |

| **Supplemental Table 2**: Total intensity due to all peptides and number of distinct peptide identities recorded for abdonimoplasty skin samples from 17 Caucasian donors | | | | | | | | |
| --- | --- | --- | --- | --- | --- | --- | --- | --- |
|  | **Epidermis membrane** | | **Dermis**  **membrane** | | **Epidermis**  **cytosol** | | **Dermis**  **cytosol** | |
| Sample | Intensity | Peptides | Intensity | Peptides | Intensity | Peptides | Intensity | Peptides |
| C24 | 2.37E+09 | 5710 | 4.08E+09 | 7730 | 1.79E+11 | 8865 | 4.94E+09 | 4518 |
| C36 | 2.99E+09 | 5441 | 4.21E+09 | 7886 | 1.77E+11 | 8970 | 2.35E+11 | 7167 |
| C37 | 3.16E+09 | 4449 | 4.07E+09 | 5876 | 1.94E+10 | 1344 | 3.51E+10 | 6709 |
| C40 | 4.75E+09 | 6630 | 1.43E+09 | 7352 | 9.56E+10 | 9203 | 4.03E+09 | 9920 |
| C43 | 4.52E+09 | 6553 | 4.56E+09 | 7814 | 6.63E+10 | 8965 | 3.3E+09 | 7163 |
| C44 | 3.22E+09 | 5459 | 2.92E+09 | 7292 | 9.39E+10 | 9316 | 6.03E+10 | 7396 |
| C46 | 3.61E+09 | 6191 | 4.82E+09 | 7924 | 3.08E+10 | 9313 | 1.9E+10 | 7396 |
| C47 | 1.93E+09 | 2577 | 1.78E+09 | 894 | 9.05E+09 | 1584 | 1E+10 | 3107 |
| C49a | 1.47E+09 | 2736 | 1.94E+09 | 892 | 9.26E+09 | 1573 | 5.92E+09 | 3032 |
| C49b | 2.9E+09 | 4406 | 6.84E+09 | 6454 | 4.15E+10 | 7898 | 1.78E+10 | 6652 |
| C50 | 4.2E+09 | 6459 | 4.56E+09 | 8143 | 1.25E+11 | 8978 | 8.63E+10 | 11069 |
| C52 | 3.89E+09 | 2979 | 1.89E+09 | 898 | 8.49E+09 | 1562 | 8.87E+10 | 12730 |
| C53 | 1.87E+09 | 5779 | 2.68E+09 | 7635 | 2.11E+10 | 8905 | 2.37E+10 | 10496 |
| C54 | 4.8E+09 | 3003 | 2.03E+09 | 910 | 9.85E+09 | 1530 | 5.97E+09 | 6118 |
| C62 | 3.71E+09 | 4328 | 1.72E+09 | 5690 | 1.64E+10 | 1517 | 1.35E+10 | 6604 |
| C64 | 3.21E+09 | 4394 | 5.56E+09 | 6559 | 1.83E+10 | 1264 | 9.45E+09 | 6069 |
| C69 | 3.19E+09 | 5203 | 3.14E+09 | 7608 | 1.39E+11 | 9099 | 1.3E+11 | 7258 |

| **Supplemental Table 3**: Human peptides recorded at unexpectedly high intensity in one or more fraction of skin | | | | |
| --- | --- | --- | --- | --- |
|  | **Percentage (%) of total protein intensity (average over all samples)** | | | |
| **Peptide** | **Epidermis membrane** | **Dermis membrane** | **Epidermis cytosol** | **Dermis**  **cytosol** |
| GITLSVRP | 37 | 28 | 0.3 | 1.1 |
| DYNVYTHSFLCYGK | 0.05 | 7.7 | 0.2 | 0.1 |
| FQIAQVVR | 0 | 0 | 3.5 | 4 |
| PEYLVSGIRTPPVR | 3 | 0 | 0 | 0 |
| LVTSELK | 0 | 0 | 1 | 1.1 |
| APECGAPALPR | 0.1 | 0.3 | 0 | 0 |
| MAAAPALK | 0 | 0 | 0.7 | 0 |
| IGSTPVLVLSR | 0.03 | 0.03 | 0.1 | 0.4 |

| **Supplemental Table 4**: The total intensity of unlabelled peptide as a percentage of the labelled peptide detected | | |
| --- | --- | --- |
| **Peptide** | **Epidermis membrane (EM) total intensity**  **Unlabelled / ^13^C_6_-labelled %** | **Dermis membrane (DM) total intensity (out of 1.71 x 10^11^)**  **Unlabelled / ^13^C_6_-labelled** |
| IGSTPVLVLSR | 1.0 | 1.0 |
| YLPNPALQR | 2.0 | 2.0 |
| ASGNLIPQEK | 0.6 | Not detected |
| EM = Epidermis membrane fraction; DC = Dermis membrane fraction from Caucasian human skin donors | | |

| **Supplemental Table 5:** Protein abundance of four fractions (17 samples each) of Caucasian human skin. Sample numbers correspond to donor age | | | | | | | | | | | | | | | | | |
| --- | --- | --- | --- | --- | --- | --- | --- | --- | --- | --- | --- | --- | --- | --- | --- | --- | --- |
|  | **Protein abundance (pmol/mg protein) of epidermis membrane (EM) fractions in 17 Caucasian human skin** | | | | | | | | | | | | | | | | |
| **Protein Name** | **EMc24** | **EMc36** | **EMc37** | **EMc40** | **EMc43** | **EMc44** | **EMc46** | **EMc47** | **EMc49a** | **EMc49b** | **EMc50** | **EMc52** | **EMc53** | **EMc54** | **EMc62** | **EMc64** | **EMc69** |
| **Trypsin 1** | 2582.0 | 1142.3 | 9384.8 | 663.7 | 159.9 | 1096.2 | 84.1 | 10489.4 | 5701.7 | 6665.2 | 422.3 | 2805.1 | 2029.7 | 1442.9 | 12398.4 | 12849.0 | 1765.2 |
| **ALBU** | 6053.9 | 6678.5 | 136.2 | 1867.5 | 2244.6 | 6575.1 | 3987.9 | 167.3 | 1658.8 | 235.7 | 4080.7 | 2646.6 | 1003.7 | 2798.4 | 110.4 | 243.3 | 1552.8 |
| **CO1A1** | 129.5 | 123.9 | 58.4 | 676.3 | 416.7 | 115.2 | 616.1 | 1404.9 | 1626.4 | 50.5 | 445.6 | 1406.1 | 883.9 | 1425.9 | 38.1 | 52.8 | 62.1 |
| **K2C1** | 612.0 | 540.9 | 354.3 | 122.5 | 148.8 | 359.2 | 295.1 | 1033.9 | 339.2 | 301.1 | 78.5 | 234.3 | 393.5 | 155.8 | 280.9 | 409.4 | 870.2 |
| **CO3A1** | 99.5 | 63.1 | 95.0 | 282.6 | 183.4 | 80.5 | 220.9 | 397.7 | 482.7 | 88.2 | 186.4 | 479.4 | 348.1 | 507.5 | 95.1 | 103.7 | 82.4 |
| **CO1A2** | 57.7 | 31.8 | 25.7 | 269.9 | 166.4 | 45.3 | 256.5 | 271.9 | 631.0 | 24.3 | 179.2 | 784.0 | 299.5 | 625.2 | 21.4 | 47.3 | 13.8 |
| **K1C10** | 296.7 | 255.3 | 282.1 | 65.3 | 85.8 | 160.0 | 162.4 | 839.2 | 224.3 | 318.5 | 71.8 | 185.6 | 245.4 | 129.2 | 130.7 | 208.5 | 359.8 |
| **LUM** | 101.3 | 91.5 | 59.8 | 330.9 | 815.4 | 84.4 | 253.6 | 55.5 | 342.3 | 77.4 | 463.3 | 552.7 | 254.3 | 805.0 | 39.1 | 68.5 | 54.3 |
| **IGKC** | 224.5 | 123.7 | 24.5 | 304.9 | 405.7 | 184.5 | 228.8 | 24.7 | 235.8 | 72.8 | 586.8 | 501.3 | 388.2 | 351.7 | 24.2 | 110.1 | 80.4 |
| **PGS2** | 82.1 | 58.2 | 30.5 | 383.0 | 433.9 | 75.5 | 307.6 | 29.4 | 351.8 | 26.5 | 566.2 | 531.6 | 261.7 | 493.8 | 31.4 | 38.1 | 51.0 |
|  | **Protein abundance (pmol/mg protein) of dermis membrane (DM) fractions in 17 Caucasian human skin** | | | | | | | | | | | | | | | | |
| **Protein Name** | **DMc24** | **DMc36** | **DMc37** | **DMc40** | **DMc43** | **DMc44** | **DMc46** | **DMc47** | **DMc49a** | **DMc49b** | **DMc50** | **DMc52** | **DMc53** | **DMc54** | **DMc62** | **DMc64** | **DMc69** |
| **Trypsin 1** | 1044.6 | 56.4 | 3554.9 | 766.9 | 9.5 | 1232.9 | 628.6 | 12356.1 | 11334.3 | 1078.5 | 219.3 | 0.0 | 1270.2 | 17206.4 | 29.3 | 2019.4 | 760.5 |
| **CO1A1** | 513.9 | 717.0 | 255.9 | 934.8 | 1394.3 | 259.9 | 814.4 | 230.8 | 202.8 | 534.6 | 754.4 | 539.5 | 429.8 | 218.9 | 94.3 | 541.8 | 353.8 |
| **ALBU** | 3757.5 | 1691.5 | 576.6 | 1460.4 | 1433.1 | 1842.4 | 991.1 | 301.9 | 492.2 | 2303.0 | 1206.6 | 714.1 | 1299.0 | 283.9 | 218.9 | 1425.0 | 2585.1 |
| **CO3A1** | 205.9 | 372.6 | 116.2 | 317.5 | 547.4 | 141.3 | 326.7 | 954.9 | 1547.0 | 168.2 | 307.7 | 1625.5 | 215.4 | 1088.5 | 69.2 | 184.6 | 148.0 |
| **K2C1** | 443.5 | 107.4 | 99.7 | 141.1 | 60.7 | 740.6 | 155.2 | 1146.5 | 803.1 | 129.1 | 38.2 | 2090.5 | 281.5 | 586.3 | 359.1 | 136.0 | 173.3 |
| **PGS2** | 344.9 | 443.6 | 61.2 | 458.8 | 309.4 | 437.3 | 540.5 | 48.7 | 82.8 | 486.9 | 485.6 | 96.6 | 421.4 | 40.6 | 2187.5 | 242.4 | 555.3 |
| **GSHB** | 470.1 | 816.3 | 7.3 | 719.2 | 200.0 | 1094.0 | 678.8 | 0.0 | 0.0 | 199.3 | 124.1 | 0.0 | 1055.1 | 0.0 | 10.0 | 420.0 | 888.4 |
| **CO1A2** | 212.8 | 399.3 | 163.5 | 340.9 | 648.2 | 132.7 | 348.1 | 58.9 | 66.1 | 297.6 | 376.0 | 77.5 | 171.7 | 46.1 | 65.8 | 429.3 | 128.1 |
| **K1C10** | 105.2 | 42.7 | 37.9 | 55.1 | 35.7 | 222.0 | 76.7 | 1236.0 | 1000.2 | 124.2 | 26.4 | 1182.9 | 126.7 | 553.2 | 185.2 | 83.2 | 70.0 |
| **LUM** | 339.0 | 286.8 | 63.5 | 448.1 | 399.3 | 336.3 | 350.6 | 60.3 | 144.7 | 599.4 | 288.3 | 250.7 | 341.3 | 49.4 | 29.5 | 280.5 | 406.6 |
|  | **Protein abundance (pmol/mg protein) of epidermis cytosol (EC) fractions in 17 Caucasian human skin** | | | | | | | | | | | | | | | | |
| **Protein Name** | **ECc24** | **ECc36** | **ECc37** | **ECc40** | **ECc43** | **ECc44** | **ECc46** | **ECc47** | **ECc49a** | **ECc49b** | **ECc50** | **ECc52** | **ECc53** | **ECc54** | **ECc62** | **ECc64** | **ECc69** |
| **ALBU** | 10589.2 | 10323 | 227.9 | 6726.8 | 9916.5 | 6752.8 | 6137.1 | 137.8 | 176.0 | 102.7 | 9856.0 | 40.1 | 9881.1 | 105.0 | 91.3 | 45.6 | 9317.1 |
| **CO1A1** | 21.3 | 24.5 | 3234.6 | 29.5 | 24.8 | 29.0 | 43.3 | 2595.8 | 2765.2 | 1927.9 | 8.0 | 3287.0 | 25.3 | 3096.3 | 2633.3 | 3629.5 | 16.4 |
| **CO1A2** | 4.8 | 4.9 | 2154.4 | 10.6 | 5.8 | 11.4 | 12.1 | 2246.7 | 2234.6 | 1363.9 | 2.4 | 2046.7 | 10.5 | 2399.0 | 1942.8 | 1955.9 | 4.6 |
| **K1C10** | 1.9 | 1.9 | 5.9 | 2.7 | 2.9 | 3.2 | 10.9 | 25.6 | 9.3 | 10.9 | 1.0 | 12.2 | 7.6 | 13.2 | 25.6 | 11.3 | 2.7 |
| **IGKC** | 555.2 | 267.7 | 64.1 | 955.2 | 1085.8 | 1870.6 | 1201.2 | 9.4 | 31.3 | 16.6 | 1157.5 | 7.8 | 849.6 | 8.5 | 8.1 | 18.5 | 657.4 |
| **IGHG1** | 343.0 | 308.7 | 105.8 | 791.3 | 1136.3 | 1912.0 | 660.8 | 16.7 | 68.4 | 277.4 | 693.5 | 16.3 | 1211.5 | 24.2 | 14.8 | 14.0 | 582.1 |
| **CO4A1** | 3.1 | 3.9 | 769.0 | 8.8 | 3.1 | 5.8 | 6.8 | 792.8 | 678.9 | 403.4 | 1.0 | 874.7 | 4.2 | 613.1 | 857.9 | 929.7 | 2.9 |
| **IGHG2** | 376.4 | 124.3 | 15.2 | 858.0 | 663.6 | 812.2 | 901.9 | 1.7 | 14.2 | 79.4 | 807.2 | 2.9 | 506.7 | 6.2 | 0.6 | 2.0 | 415.2 |
| **K2C1** | 5.3 | 4.7 | 21.4 | 11.5 | 10.7 | 25.2 | 40.0 | 14.4 | 11.7 | 9.2 | 4.0 | 13.7 | 18.1 | 7.3 | 28.2 | 20.5 | 8.2 |
| **TRFE** | 291.6 | 804.7 | 8.7 | 383.8 | 352.3 | 288.6 | 295.3 | 10.6 | 13.8 | 168.4 | 633.4 | 8.3 | 380.7 | 11.4 | 5.1 | 2.4 | 586.5 |
|  | **Protein abundance (pmol/mg protein) of dermis cytosol (DC) fractions in 17 Caucasian human skin** | | | | | | | | | | | | | | | | |
| **Protein Name** | **DCc24** | **DCc36** | **DCc37** | **DCc40** | **DCc43** | **DCc44** | **DCc46** | **DCc47** | **DCc49a** | **DCc49b** | **DCc50** | **DCc52** | **DCc53** | **DCc54** | **DCc62** | **DCc64** | **DCc69** |
| **ALBU** | 10402.7 | 8239.5 | 2454.9 | 8597.4 | 3447.0 | 8013.2 | 8042.0 | 6428.7 | 8688.6 | 2178.3 | 5675.5 | 4872.9 | 8914.7 | 3037.4 | 2021.5 | 6274.1 | 7086.8 |
| **IGKC** | 693.0 | 394.4 | 854.6 | 760.2 | 173.4 | 1369.6 | 845.3 | 782.7 | 1038.3 | 361.5 | 651.4 | 443.3 | 436.1 | 351.1 | 331.9 | 859.3 | 738.7 |
| **IGHG1** | 795.5 | 393.6 | 804.4 | 526.6 | 457.4 | 1398.1 | 876.0 | 762.9 | 565.0 | 197.5 | 441.3 | 615.1 | 360.1 | 262.8 | 177.9 | 278.2 | 714.8 |
| **IGHG2** | 324.8 | 130.2 | 429.7 | 543.2 | 99.9 | 632.8 | 608.3 | 944.6 | 1306.1 | 307.1 | 503.5 | 313.6 | 176.9 | 380.9 | 186.9 | 576.3 | 474.1 |
| **PAR14** | 0.0 | 977.9 | 424.9 | 459.3 | 0.0 | 723.4 | 765.2 | 0.0 | 0.0 | 253.9 | 918.9 | 546.6 | 742.4 | 0.0 | 181.0 | 0.5 | 1260.2 |
| **TRFE** | 209.2 | 484.6 | 197.3 | 543.4 | 165.0 | 209.1 | 208.9 | 935.8 | 673.9 | 242.2 | 283.5 | 185.4 | 208.5 | 484.8 | 164.7 | 306.1 | 403.4 |
| **LUM** | 110.7 | 155.0 | 45.0 | 85.2 | 23.3 | 115.8 | 94.4 | 205.0 | 212.7 | 40.7 | 150.3 | 384.7 | 94.3 | 94.3 | 41.1 | 76.6 | 99.4 |
| **FLNA** | 10.1 | 11.4 | 15.5 | 20.6 | 1112.6 | 14.9 | 19.4 | 95.7 | 16.9 | 13.0 | 802.2 | 870.7 | 19.2 | 81.9 | 37.4 | 13.5 | 6.3 |
| **PGS2** | 95.7 | 29.6 | 114.4 | 109.5 | 35.8 | 100.9 | 135.5 | 570.5 | 196.3 | 140.4 | 42.3 | 211.3 | 110.6 | 540.3 | 248.5 | 36.6 | 58.5 |
| **IGLL5** | 76.3 | 72.0 | 148.3 | 844.3 | 54.9 | 350.8 | 180.6 | 122.8 | 100.0 | 46.5 | 114.0 | 184.7 | 450.2 | 103.7 | 36.0 | 60.0 | 165.6 |

| **Supplemental Table 6:** SLC transporter peptides found in cytosolic fractions of Caucasian human skin | | | |
| --- | --- | --- | --- |
| **Protein** | **Peptides** | **Epidermis cytosol (EC)** | **Dermis cytosol (DC)** |
| ADT2 (SLC25A5)/ ADT3 (SLC25A6) | DFLAGGVAAAISK | Range 0 – 0.34 pmol/mg total protein | Range 0 – 0.06 pmol/mg total protein |
|  | GMLPDPK |  |  |
|  | TAVAPIER |  |  |
|  | GLGDCLVK |  |  |
|  | LLLQVQHASK |  |  |
|  | GAWSNVLR |  |  |
| S12A2 | AAAAAAAAAAAAAAAGAGAGAK | No significant abundance | Range 0 – 0.65 pmol/mg total protein |
|  | FQVDLVSENAGR |  |  |
|  | VELPGTAVPSVPEDAAPASR |  |  |
|  | QTPADGEASGESEPAK |  |  |
|  | DLPPILLVR |  |  |
| MPCP (SLC25A3) | IQTQPGYANTLR | No significant abundance | Range 0 – 0.06 pmole /mg total protein |
|  | GVAPLWMR |  |  |
|  | GIFNGFSVTLK |  |  |
|  | TVEALYK |  |  |

EC = Epidermis cytosolic fraction; DC = Dermis cytosolic fraction from Caucasian human skin donors

| **Supplemental Table 7:** Protein abundance of transporters in dermis and epidermis membrane fractions from 17 human donors, expressed as pmol/mg protein. Sample numbers correspond to donor age | | | | | | | | | | | | | | | | | | | | |
| --- | --- | --- | --- | --- | --- | --- | --- | --- | --- | --- | --- | --- | --- | --- | --- | --- | --- | --- | --- | --- |
| **Protein** | **DMc24** | **DMc36** | **DMc37** | **DMc40** | **DMc43** | **DMc44** | **DMc46** | **DMc47** | **DMc49a** | **DMc49b** | **DMc50** | **DMc52** | **DMc53** | **DMc54** | **DMc62** | **DMc64** | **DMc69** | **Mean** | **SD** | **% CV** |
| **SLC12A2/S12A2** | 0.23 | 0.49 | 0.0 | 0.12 | 0.03 | 0.11 | 0.55 | 0.0 | 0.0 | 0.0 | 1.93 | 0.0 | 0.61 | 0.0 | 0.02 | 0.0 | 0.04 | 0.24 | 0.48 | 198.4 |
| **SLC15A1/S15A1** | 4.21 | 5.45 | 4.05 | 2.87 | 1.40 | 7.68 | 4.70 | 53.7 | 38.1 | 2.39 | 1.46 | 78.9 | 4.06 | 54.9 | 5.05 | 2.95 | 6.51 | 16.4 | 24.1 | 147.0 |
| **SLC16A1/MOT1** | 4.69 | 5.25 | 7.40 | 7.35 | 2.60 | 6.59 | 3.73 | 21.4 | 28.4 | 4.49 | 1.82 | 27.5 | 7.91 | 20.5 | 5.56 | 5.86 | 7.52 | 9.92 | 8.66 | 87.3 |
| **SLC22A1/S22A1** | 2.20 | 3.08 | 9.57 | 2.34 | 1.52 | 6.26 | 4.06 | 63.8 | 49.1 | 3.18 | 0.77 | 81.4 | 2.90 | 39.7 | 6.98 | 5.79 | 3.84 | 16.9 | 25.2 | 149.4 |
| **SLC22A3/S22A3** | 4.89 | 8.16 | 13.4 | 6.19 | 2.59 | 17.8 | 9.48 | 7.61 | 16.4 | 11.2 | 1.94 | 0.0 | 5.90 | 13.1 | 21.1 | 10.1 | 11.2 | 9.47 | 5.80 | 60.8 |
| **SLC22A7/S22A7** | 7.15 | 8.28 | 7.39 | 9.41 | 2.89 | 19.8 | 7.59 | 0.0 | 0.0 | 4.57 | 2.18 | 0.0 | 9.50 | 0.0 | 11.3 | 9.20 | 11.8 | 6.53 | 5.37 | 82.3 |
| **SLC22A9/S22A9** | 11.3 | 11.3 | 10.3 | 10.5 | 4.13 | 18.4 | 8.45 | 13.6 | 37.4 | 4.07 | 2.55 | 17.7 | 14.3 | 16.2 | 11.2 | 12.8 | 17.1 | 13.0 | 7.90 | 60.0 |
| **SLC25A1/TXTP** | 0.02 | 0.0 | 0.03 | 0.01 | 0.02 | 0.05 | 0.09 | 0.0 | 0.0 | 0.01 | 0.09 | 0.0 | 0.01 | 0.0 | 0.0 | 0.04 | 0.03 | 0.02 | 0.03 | 124.8 |
| **SLC25A11/M2OM** | 0.07 | 0.02 | 0.01 | 0.05 | 0.06 | 0.0 | 0.10 | 0.0 | 0.0 | 0.0 | 0.16 | 0.0 | 0.07 | 0.0 | 0.0 | 0.0 | 0.01 | 0.03 | 0.05 | 143.1 |
| **SLC25A12/CMC1** | 0.11 | 0.04 | 0.0 | 0.04 | 0.04 | 0.01 | 0.04 | 0.0 | 0.0 | 0.0 | 0.36 | 0.0 | 0.06 | 0.0 | 0.0 | 0.0 | 0.03 | 0.04 | 0.09 | 202.8 |
| **SLC25A24/SCMC1** | 0.02 | 0.06 | 0.40 | 0.02 | 0.10 | 0.01 | 0.16 | 0.0 | 0.0 | 0.01 | 0.33 | 0.0 | 0.10 | 0.0 | 0.08 | 0.0 | 0.0 | 0.08 | 0.12 | 157.2 |
| **SLC25A3/MPCP** | 1.41 | 2.04 | 0.77 | 0.81 | 1.24 | 1.19 | 2.82 | 0.0 | 0.0 | 0.12 | 3.10 | 0.0 | 11.8 | 0.0 | 0.42 | 0.19 | 1.41 | 1.61 | 2.81 | 174.5 |
| **SLC25A4/ADT1** | 0.56 | 2.38 | 0.0 | 0.58 | 0.50 | 2.93 | 0.26 | 0.0 | 0.0 | 0.0 | 0.70 | 0.0 | 0.40 | 0.0 | 1.38 | 0.39 | 0.57 | 0.63 | 0.85 | 135.8 |
| **SLC25A5/ADT2** | 21.3 | 6.38 | 3.38 | 7.70 | 3.75 | 32.3 | 13.8 | 0.0 | 0.0 | 1.86 | 3.58 | 0.0 | 14.1 | 0.0 | 0.46 | 3.13 | 8.40 | 7.07 | 8.90 | 125.6 |
| **SLC25A6/ADT3** | 0.0 | 0.02 | 0.11 | 0.0 | 0.0 | 0.38 | 5.09 | 0.09 | 0.07 | 1.36 | 2.95 | 0.0 | 3.35 | 0.0 | 0.23 | 1.49 | 1.80 | 1.00 | 1.51 | 151.5 |
| **SLC2A1/GTR1** | 0.14 | 0.11 | 0.09 | 0.03 | 0.11 | 0.04 | 0.14 | 0.0 | 0.0 | 0.05 | 0.50 | 0.0 | 0.23 | 0.0 | 0.07 | 0.12 | 0.11 | 0.10 | 0.12 | 117.8 |
| **SLC35F3/S35F3** | 0.12 | 0.19 | 0.0 | 0.08 | 0.08 | 0.01 | 0.29 | 0.0 | 0.0 | 0.05 | 0.17 | 0.0 | 0.27 | 0.0 | 0.0 | 0.02 | 0.16 | 0.08 | 0.10 | 116.5 |
| **SLC44A1/CTL1** | 0.08 | 0.0 | 0.0 | 0.01 | 0.0 | 0.04 | 0.0 | 0.0 | 0.0 | 0.01 | 0.02 | 0.0 | 0.0 | 0.0 | 0.0 | 0.02 | 0.01 | 0.01 | 0.02 | 186.8 |
| **SLC44A2/CTL2** | 0.0 | 0.12 | 0.02 | 0.01 | 0.03 | 0.08 | 0.17 | 0.0 | 0.0 | 0.0 | 0.08 | 0.0 | 0.01 | 0.0 | 0.02 | 0.01 | 0.13 | 0.04 | 0.05 | 136.6 |
| **SLC47A1/S47A1** | 9.59 | 13.7 | 30.1 | 13.7 | 5.39 | 24.5 | 11.9 | 67.0 | 78.7 | 14.4 | 4.0 | 69.9 | 18.0 | 64.6 | 38.3 | 21.3 | 16.0 | 29.5 | 24.8 | 84.1 |
| **SLC4A1/B3AT** | 0.44 | 0.50 | 1.20 | 0.10 | 0.30 | 0.09 | 0.70 | 0.0 | 0.0 | 0.17 | 4.92 | 0.0 | 0.75 | 0.0 | 0.85 | 0.58 | 0.13 | 0.63 | 1.16 | 184.0 |
| **SLC51A/OSTA** | 9.79 | 12.7 | 28.4 | 0.29 | 1.64 | 3.54 | 0.87 | 0.0 | 0.0 | 13.4 | 3.71 | 0.0 | 14.5 | 0.0 | 36.8 | 10.9 | 16.4 | 8.99 | 10.8 | 119.6 |
| **SLC51B/OSTB** | 3.54 | 5.11 | 13.0 | 4.51 | 2.09 | 10.5 | 4.98 | 2.54 | 71.4 | 6.92 | 1.31 | 1.07 | 5.58 | 102 | 15.3 | 7.41 | 6.45 | 15.5 | 27.6 | 177.9 |
| **SLCO1A2/SO1A2** | 8.29 | 13.5 | 6.57 | 9.39 | 3.88 | 35.6 | 17.2 | 0.0 | 0.0 | 3.22 | 3.40 | 0.0 | 12.3 | 0.0 | 27.6 | 4.04 | 17.8 | 9.57 | 10.2 | 106.7 |
| **SLCO1B1/SO1B1** | 1.91 | 4.75 | 11.2 | 2.06 | 1.50 | 12.5 | 4.49 | 40.6 | 38.2 | 0.21 | 0.99 | 72.8 | 5.38 | 50.0 | 15.0 | 5.37 | 4.06 | 15.9 | 21.2 | 133.3 |
| **SLCO2B1/SO2B1** | 12.4 | 18.5 | 8.88 | 11.3 | 4.34 | 33.5 | 13.4 | 162 | 197 | 3.98 | 2.59 | 136 | 14.2 | 148 | 16.8 | 11.1 | 22.2 | 48.0 | 65.8 | 137.1 |
| **SLCO4C1/SO4C1** | 2.77 | 4.96 | 13.6 | 3.46 | 1.41 | 12.0 | 6.07 | 74.5 | 42.3 | 6.86 | 1.17 | 110 | 6.18 | 65.8 | 16.0 | 9.07 | 6.34 | 22.5 | 31.6 | 140.6 |
| **Protein** | **EMc24** | **EMc36** | **EMc37** | **EMc40** | **EMc43** | **EMc44** | **EMc46** | **EMc47** | **EMc49a** | **EMc49b** | **EMc50** | **EMc52** | **EMc53** | **EMc54** | **EMc62** | **EMc64** | **EMc69** | **Mean** | **SD** | **% CV** |
| **SLC12A2/S12A2** | 0.16 | 0.41 | 0.11 | 2.28 | 1.61 | 0.70 | 1.49 | 0.0 | 0.0 | 0.09 | 0.77 | 0.0 | 0.39 | 0.0 | 0.01 | 0.10 | 0.67 | 0.50 | 0.70 | 131.3 |
| **SLC15A1/S15A1** | 8.39 | 10.4 | 13.6 | 2.85 | 4.04 | 1.19 | 6.15 | 33.9 | 24.1 | 9.99 | 1.95 | 8.41 | 7.53 | 4.50 | 0.13 | 13.3 | 2.23 | 9.00 | 8.70 | 97.3 |
| **SLC16A1/MOT1** | 10.4 | 8.72 | 13.7 | 5.22 | 3.46 | 4.09 | 6.62 | 14.7 | 12.2 | 28.2 | 5.48 | 5.61 | 20.7 | 2.27 | 8.89 | 1.58 | 8.71 | 9.40 | 6.90 | 73.3 |
| **SLC22A1/S22A1** | 5.37 | 8.20 | 23.4 | 1.63 | 1.85 | 15.1 | 5.53 | 29.5 | 21.5 | 24.1 | 1.29 | 7.70 | 6.09 | 4.20 | 28.2 | 33.1 | 11.6 | 13.4 | 10.9 | 81.0 |
| **SLC22A3/S22A3** | 12.5 | 17.4 | 47.6 | 5.38 | 8.50 | 11.4 | 12.9 | 8.28 | 4.68 | 39.6 | 4.54 | 3.94 | 14.5 | 1.19 | 39.3 | 46.0 | 20.6 | 17.5 | 15.6 | 88.8 |
| **SLC22A7/S22A7** | 17.3 | 23.4 | 22.5 | 4.03 | 4.83 | 20.1 | 11.9 | 0.0 | 0.0 | 32.4 | 4.00 | 0.0 | 19.8 | 0.0 | 20.8 | 23.8 | 22.4 | 13.4 | 10.8 | 80.6 |
| **SLC22A9/S22A9** | 25.8 | 19.1 | 7.47 | 6.71 | 4.47 | 16.2 | 10.8 | 11.3 | 9.69 | 21.9 | 5.62 | 8.49 | 28.4 | 1.66 | 31.5 | 31.5 | 71.9 | 18.4 | 16.9 | 91.8 |
| **SLC25A1/TXTP** | 0.0 | 0.0 | 0.03 | 0.04 | 0.20 | 0.0 | 0.01 | 0.0 | 0.0 | 0.0 | 0.0 | 0.0 | 0.01 | 0.0 | 0.08 | 0.06 | 0.01 | 0.0 | 0.10 | 196.6 |
| **SLC25A11/M2OM** | 0.01 | 0.0 | 0.0 | 0.03 | 0.17 | 0.0 | 0.01 | 0.0 | 0.0 | 0.20 | 0.08 | 0.0 | 0.0 | 0.0 | 0.0 | 0.0 | 0.0 | 0.0 | 0.10 | 211.1 |
| **SLC25A12/CMC1** | 0.01 | 0.0 | 0.0 | 0.10 | 0.01 | 0.01 | 0.03 | 0.0 | 0.0 | 0.0 | 0.01 | 0.0 | 0.01 | 0.0 | 0.0 | 0.0 | 0.13 | 0.0 | 0.0 | 206.3 |
| **SLC25A24/SCMC1** | 0.0 | 0.0 | 0.01 | 0.04 | 0.19 | 0.02 | 0.01 | 0.0 | 0.0 | 0.01 | 0.16 | 0.0 | 0.0 | 0.0 | 0.01 | 0.04 | 0.0 | 0.0 | 0.10 | 197.0 |
| **SLC25A3/MPCP** | 0.98 | 1.44 | 0.97 | 1.81 | 3.42 | 0.75 | 2.69 | 0.0 | 0.0 | 0.81 | 2.17 | 0.02 | 1.29 | 0.0 | 3.48 | 0.17 | 2.67 | 1.30 | 1.20 | 89.5 |
| **SLC25A4/ADT1** | 4.94 | 4.38 | 0.0 | 0.46 | 0.67 | 2.92 | 1.38 | 0.0 | 0.0 | 1.48 | 0.60 | 0.0 | 2.53 | 0.0 | 0.0 | 5.22 | 3.66 | 1.70 | 1.90 | 114.0 |
| **SLC25A5/ADT2** | 14.9 | 10.4 | 8.02 | 6.42 | 6.05 | 6.81 | 18.2 | 0.20 | 0.20 | 2.20 | 5.55 | 0.08 | 11.3 | 0.07 | 5.23 | 4.76 | 39.8 | 8.20 | 9.70 | 117.1 |
| **SLC25A6/ADT3** | 2.78 | 0.36 | 0.0 | 0.28 | 5.30 | 4.09 | 0.0 | 0.0 | 0.0 | 0.0 | 0.13 | 0.01 | 0.16 | 0.0 | 0.0 | 0.0 | 0.0 | 0.80 | 1.60 | 211.8 |
| **SLC47A1/S47A1** | 29.9 | 27.0 | 128 | 27.2 | 45.0 | 21.5 | 16.5 | 36.7 | 23.3 | 115 | 9.0 | 10.0 | 49.2 | 4.9 | 92.0 | 103 | 27.3 | 45.0 | 39.2 | 87.1 |
| **SLC4A1/B3AT** | 0.03 | 0.01 | 0.06 | 0.02 | 0.03 | 0.03 | 0.04 | 0.0 | 0.0 | 0.33 | 0.06 | 0.02 | 0.02 | 0.0 | 0.01 | 0.04 | 0.0 | 0.0 | 0.10 | 186.7 |
| **SLC51A/OSTA** | 20.9 | 8.53 | 0.0 | 5.47 | 6.17 | 26.1 | 0.08 | 0.0 | 0.0 | 0.19 | 2.34 | 0.0 | 0.32 | 0.0 | 66.0 | 87.2 | 31.1 | 15.0 | 25.5 | 170.3 |
| **SLC51B/OSTB** | 5.82 | 8.93 | 49.8 | 3.45 | 2.48 | 2.04 | 5.95 | 0.01 | 21.8 | 36.1 | 1.71 | 10.5 | 7.37 | 0.41 | 29.5 | 44.8 | 2.13 | 13.7 | 16.4 | 119.6 |
| **SLCO1A2/SO1A2** | 20.9 | 39.9 | 133 | 6.53 | 5.91 | 24.9 | 20.9 | 0.0 | 0.0 | 95.3 | 5.96 | 0.0 | 25.3 | 0.0 | 68.4 | 83.2 | 27.3 | 32.8 | 39.3 | 119.8 |
| **SLCO1B1/SO1B1** | 7.31 | 15.4 | 0.03 | 2.74 | 0.60 | 13.1 | 7.75 | 22.9 | 18.3 | 0.20 | 1.32 | 5.14 | 5.37 | 2.24 | 16.5 | 25.9 | 5.61 | 8.90 | 8.30 | 93.5 |
| **SLCO1B3/SO1B3** | 13.0 | 10.1 | 0.40 | 2.97 | 0.29 | 0.12 | 7.45 | 0.0 | 0.0 | 1.24 | 2.61 | 0.0 | 11.3 | 0.0 | 13.0 | 8.60 | 11.2 | 4.80 | 5.30 | 108.6 |
| **SLCO2B1/SO2B1** | 40.0 | 49.0 | 42.9 | 8.77 | 9.49 | 29.1 | 29.2 | 80.6 | 57.7 | 44.1 | 9.00 | 36.1 | 40.9 | 9.49 | 0.0 | 15.1 | 33.4 | 31.5 | 21.1 | 67.2 |
| **SLCO4C1/SO4C1** | 8.36 | 15.1 | 56.4 | 2.84 | 2.23 | 12.3 | 9.05 | 36.8 | 27.4 | 37.3 | 0.87 | 9.10 | 9.41 | 4.69 | 37.0 | 65.4 | 16.5 | 20.6 | 19.5 | 94.6 |

Sample identifiers indicate tissue and donor information: **D** = Dermis, **E** = Epidermis, **M** = Membrane fraction, **c** = Caucasian

| **Supplemental Table 8:** Protein abundance of enzymes in dermis and epidermis membrane fractions from 17 human donors, expressed as pmol/mg protein. Sample numbers correspond to donor age | | | | | | | | | | | | | | | | | | | | |
| --- | --- | --- | --- | --- | --- | --- | --- | --- | --- | --- | --- | --- | --- | --- | --- | --- | --- | --- | --- | --- |
| **Enzyme** | **DMc24** | **DMc36** | **DMc37** | **DMc40** | **DMc43** | **DMc44** | **DMc46** | **DMc47** | **DMc49a** | **DMc49b** | **DMc50** | **DMc52** | **DMc53** | **DMc54** | **DMc62** | **DMc64** | **DMc69** | **Mean** | **SD** | **% CV** |
| **AKR1A1** | 0.10 | 0.16 | 0.0 | 0.12 | 0.19 | 0.02 | 0.22 | 0.0 | 0.0 | 0.05 | 0.32 | 0.0 | 0.09 | 0.0 | 0.0 | 0.04 | 0.03 | 0.10 | 0.10 | 120.7 |
| **AKR1B1** | 0.07 | 0.19 | 0.0 | 0.04 | 0.02 | 0.03 | 0.26 | 0.0 | 0.0 | 0.04 | 0.29 | 0.0 | 0.07 | 0.0 | 0.03 | 0.0 | 0.0 | 0.10 | 0.10 | 152.6 |
| **AKR1C4** | 0.18 | 0.27 | 0.0 | 0.33 | 0.29 | 0.16 | 0.70 | 0.0 | 0.0 | 0.14 | 0.96 | 0.0 | 0.19 | 0.0 | 0.0 | 0.01 | 0.21 | 0.20 | 0.30 | 131.9 |
| **ALDH1A1** | 1.43 | 1.48 | 0.15 | 1.16 | 0.90 | 1.17 | 1.50 | 0.0 | 0.0 | 0.66 | 1.57 | 0.0 | 1.26 | 0.0 | 0.05 | 0.87 | 1.09 | 0.80 | 0.60 | 78.8 |
| **ALDH1B1** | 0.03 | 0.02 | 0.0 | 0.09 | 0.02 | 0.0 | 0.05 | 0.0 | 0.0 | 0.01 | 0.03 | 0.0 | 0.04 | 0.0 | 0.0 | 0.0 | 0.02 | 0.0 | 0.0 | 134.7 |
| **ALDH2** | 1.18 | 0.81 | 0.27 | 1.88 | 1.15 | 0.36 | 2.05 | 0.0 | 0.0 | 1.58 | 2.81 | 0.0 | 1.02 | 0.0 | 1.46 | 1.31 | 0.73 | 1.00 | 0.80 | 84.5 |
| **ALDH3A1** | 0.02 | 0.02 | 0.0 | 0.0 | 0.0 | 0.02 | 0.0 | 0.0 | 0.0 | 0.0 | 0.0 | 0.0 | 0.0 | 0.0 | 0.0 | 0.0 | 0.06 | 0.0 | 0.0 | 222.7 |
| **ALDH3A2** | 0.55 | 0.79 | 0.30 | 1.03 | 0.84 | 0.51 | 1.57 | 0.0 | 0.0 | 0.14 | 1.70 | 0.0 | 1.05 | 0.0 | 0.23 | 0.24 | 0.58 | 0.60 | 0.50 | 95.9 |
| **ALDH4A1** | 1.65 | 3.49 | 0.28 | 3.10 | 3.34 | 0.65 | 6.78 | 0.0 | 0.0 | 1.40 | 4.33 | 0.0 | 2.58 | 0.0 | 0.09 | 1.04 | 1.90 | 1.80 | 1.90 | 106.6 |
| **ALDH6A1** | 1.12 | 4.58 | 0.02 | 1.92 | 1.10 | 9.78 | 5.68 | 0.0 | 0.0 | 0.76 | 2.29 | 0.0 | 2.22 | 0.0 | 0.05 | 2.34 | 5.72 | 2.20 | 2.70 | 124.4 |
| **ALDH7A1** | 0.04 | 0.0 | 0.0 | 0.0 | 0.06 | 0.0 | 0.0 | 0.0 | 0.0 | 0.0 | 0.19 | 0.0 | 0.08 | 0.0 | 0.0 | 0.0 | 0.0 | 0.0 | 0.0 | 228.7 |
| **ALDH9A1** | 1.13 | 0.87 | 3.57 | 1.69 | 1.96 | 0.59 | 1.48 | 0.0 | 0.0 | 0.51 | 2.00 | 0.0 | 1.57 | 0.0 | 0.17 | 0.78 | 0.65 | 1.00 | 1.00 | 96.5 |
| **AOC3** | 9.85 | 7.73 | 1.70 | 8.39 | 5.01 | 8.52 | 15.1 | 0.02 | 0.35 | 5.12 | 17.0 | 0.03 | 9.30 | 0.02 | 3.01 | 2.37 | 17.9 | 6.60 | 5.90 | 90.5 |
| **CES1** | 0.35 | 0.43 | 0 | 0.12 | 0.16 | 0.2 | 0.87 | 0 | 0 | 0.34 | 0.29 | 0 | 0.42 | 0 | 0.04 | 0.05 | 0.04 | 0.19 | 0.23 | 121 |
| **COMT** | 0.27 | 0.15 | 0.19 | 0.19 | 0.43 | 0.09 | 0.29 | 0.0 | 0.0 | 0.06 | 1.78 | 0.0 | 0.20 | 0.0 | 0.32 | 0.03 | 0.06 | 0.20 | 0.40 | 175.0 |
| **CYP4F2** | 0.0 | 0.0 | 0.0 | 0.0 | 0.0 | 0.03 | 0.02 | 0.0 | 0.0 | 0.01 | 0.03 | 0.0 | 0.01 | 0.0 | 0.0 | 0.0 | 0.0 | 0.0 | 0.0 | 180.9 |
| **EPHX1** | 25.1 | 43.1 | 11.3 | 35.4 | 12.4 | 75.2 | 44.3 | 0.0 | 0.0 | 2.91 | 11.7 | 0.0 | 46.7 | 0.0 | 11.9 | 18.6 | 45.9 | 22.6 | 22.2 | 98.0 |
| **ESD** | 0.54 | 0.94 | 0.34 | 0.72 | 0.71 | 0.37 | 0.70 | 0.0 | 0.0 | 0.06 | 1.24 | 0.0 | 0.83 | 0.0 | 0.36 | 0.55 | 0.36 | 0.50 | 0.40 | 82.1 |
| **GPX1** | 0.03 | 0.06 | 0.0 | 0.02 | 0.01 | 0.0 | 0.05 | 0.0 | 0.0 | 0.0 | 0.04 | 0.0 | 0.01 | 0.0 | 0.0 | 0.01 | 0.01 | 0.0 | 0.0 | 137.3 |
| **GPX3** | 1.97 | 0.58 | 0.08 | 1.98 | 0.57 | 0.46 | 0.69 | 0.0 | 0.0 | 0.18 | 5.41 | 0.0 | 1.06 | 0.0 | 0.06 | 0.20 | 1.97 | 0.90 | 1.40 | 152.8 |
| **GSTM2** | 0.0 | 0.06 | 0.0 | 0.0 | 0.0 | 0.01 | 0.0 | 0.0 | 0.0 | 0.0 | 0.10 | 0.0 | 0.0 | 0.0 | 0.14 | 0.0 | 0.07 | 0.0 | 0.0 | 192.8 |
| **GSTM3** | 8.41 | 16.5 | 6.42 | 8.84 | 9.01 | 9.38 | 14.9 | 0.0 | 0.0 | 5.63 | 2.91 | 0.0 | 13.9 | 0.0 | 9.38 | 5.96 | 22.7 | 7.90 | 6.50 | 82.2 |
| **GSTM4** | 0.15 | 0.13 | 0.12 | 0.0 | 0.02 | 0.21 | 0.26 | 0.0 | 0.0 | 0.13 | 1.15 | 0.0 | 0.06 | 0.0 | 0.18 | 0.22 | 0.14 | 0.20 | 0.30 | 165.1 |
| **GSTP1 rs1695** | 0.0 | 0.0 | 0.18 | 0.0 | 0.01 | 1.19 | 0.53 | 0.0 | 0.0 | 0.08 | 0.02 | 0.0 | 0.0 | 0.0 | 0.0 | 0.09 | 0.74 | 0.20 | 0.30 | 201.8 |
| **GSTP1** | 6.30 | 3.29 | 1.15 | 2.82 | 3.51 | 0.0 | 3.41 | 0.0 | 0.0 | 2.52 | 6.96 | 0.0 | 3.89 | 0.0 | 0.0 | 2.71 | 1.26 | 2.20 | 2.20 | 99.5 |
| **LTA4H** | 0.02 | 0.06 | 0.09 | 0.06 | 0.0 | 0.03 | 0.05 | 0.0 | 0.0 | 0.01 | 0.32 | 0.0 | 0.05 | 0.0 | 0.01 | 0.15 | 0.06 | 0.10 | 0.10 | 148.9 |
| **MAOA** | 0.07 | 0.19 | 0.05 | 0.05 | 0.04 | 0.01 | 0.38 | 0.0 | 0.0 | 0.01 | 0.16 | 0.0 | 0.09 | 0.0 | 0.22 | 0.02 | 0.06 | 0.10 | 0.10 | 130.2 |
| **MAOB** | 0.31 | 0.41 | 0.09 | 0.27 | 0.22 | 0.14 | 0.98 | 0.0 | 0.0 | 0.02 | 0.61 | 0.0 | 0.31 | 0.0 | 0.26 | 0.13 | 0.23 | 0.20 | 0.30 | 109.3 |
| **Enzyme** | **EMc24** | **EMc36** | **EMc37** | **EMc40** | **EMc43** | **EMc44** | **EMc46** | **EMc47** | **EMc49a** | **EMc49b** | **EMc50** | **EMc52** | **EMc53** | **EMc54** | **EMc62** | **EMc64** | **EMc69** | **Mean** | **SD** | **% CV** |
| **AKR1A1** | 0.01 | 0.0 | 0.03 | 0.19 | 0.86 | 0.05 | 0.19 | 0.0 | 0.0 | 0.0 | 0.10 | 0.0 | 0.01 | 0.0 | 0.15 | 0.0 | 0.04 | 0.10 | 0.20 | 217.1 |
| **AKR1C4** | 0.03 | 0.12 | 0.0 | 0.33 | 0.27 | 0.0 | 0.17 | 0.0 | 0.0 | 0.0 | 0.06 | 0.0 | 0.10 | 0.0 | 0.0 | 0.0 | 0.0 | 0.10 | 0.10 | 162.7 |
| **ALDH16A1** | 0.01 | 0.01 | 0.0 | 0.30 | 0.16 | 0.02 | 0.12 | 0.0 | 0.0 | 0.0 | 0.05 | 0.0 | 0.0 | 0.0 | 0.0 | 0.01 | 0.11 | 0.0 | 0.10 | 177.3 |
| **ALDH1A1** | 0.69 | 0.19 | 0.08 | 1.4 | 1.49 | 0.19 | 0.78 | 0.02 | 0.38 | 0.5 | 1.37 | 1.07 | 0.32 | 0.94 | 0.02 | 0.05 | 0.06 | 0.56 | 0.52 | 93.0 |
| **ALDH2** | 0.37 | 0.35 | 0.26 | 5.21 | 2.86 | 0.28 | 3.38 | 0.30 | 0.80 | 1.00 | 2.53 | 2.63 | 1.54 | 1.20 | 1.71 | 0.73 | 3.43 | 1.70 | 1.40 | 85.7 |
| **ALDH3A1** | 0.0 | 0.0 | 0.0 | 0.0 | 0.0 | 0.0 | 0.0 | 0.02 | 0.06 | 0.0 | 0.0 | 0.16 | 0.0 | 0.21 | 0.0 | 0.0 | 0.0 | 0.0 | 0.10 | 234.8 |
| **ALDH3A2** | 1.89 | 2.95 | 0.14 | 2.97 | 1.81 | 0.51 | 4.11 | 0.0 | 0.0 | 0.64 | 1.42 | 0.02 | 2.58 | 0.0 | 0.29 | 0.16 | 0.28 | 1.20 | 1.30 | 113.4 |
| **ALDH7A1** | 0.01 | 0.01 | 0.07 | 0.41 | 0.02 | 0.22 | 0.13 | 0.0 | 0.0 | 0.0 | 0.01 | 0.0 | 0.01 | 0.0 | 0.01 | 0.0 | 0.20 | 0.10 | 0.10 | 176.2 |
| **ALDH9A1** | 0.72 | 0.23 | 0.10 | 3.19 | 2.88 | 0.56 | 1.55 | 0.25 | 0.24 | 0.04 | 9.65 | 1.45 | 5.16 | 0.86 | 0.71 | 0.04 | 0.74 | 1.70 | 2.50 | 148.4 |
| **AOC3** | 0.59 | 0.64 | 3.49 | 1.57 | 1.79 | 0.50 | 1.63 | 0.0 | 0.0 | 0.45 | 1.04 | 0.0 | 0.48 | 0.0 | 1.91 | 1.56 | 1.09 | 1.00 | 0.90 | 94.4 |
| **CES1** | 34.5 | 30.9 | 58.9 | 8.79 | 11.0 | 29.7 | 10.9 | 45.8 | 47.5 | 95.20 | 14.0 | 26.3 | 51.9 | 12.1 | 54.1 | 40.0 | 27.2 | 35.2 | 22.5 | 64.0 |
| **COMT** | 0.07 | 0.0 | 0.01 | 1.63 | 3.82 | 0.05 | 0.05 | 0.0 | 0.0 | 0.17 | 0.87 | 0.0 | 0.0 | 0.0 | 0.23 | 0.12 | 0.02 | 0.40 | 1.00 | 235.3 |
| **EPHX1** | 52.3 | 88.6 | 29.5 | 17.9 | 19.4 | 65.8 | 42.3 | 48.2 | 55.7 | 49.24 | 17.9 | 27.4 | 72.5 | 12.7 | 24.4 | 20.6 | 28.5 | 39.6 | 22.1 | 55.8 |
| **GPX3** | 2.04 | 0.43 | 0.05 | 8.94 | 6.25 | 0.62 | 1.77 | 0.0 | 0.0 | 0.15 | 8.91 | 0.08 | 0.84 | 0.14 | 0.17 | 0.08 | 1.60 | 1.90 | 3.10 | 161.8 |
| **GSTM1** | 0.0 | 0.0 | 0.01 | 0.0 | 0.0 | 0.0 | 0.0 | 0.0 | 0.0 | 0.01 | 0.0 | 0.0 | 0.09 | 0.0 | 0.03 | 0.02 | 0.0 | 0.0 | 0.0 | 239.0 |
| **GSTM2** | 0.03 | 0.01 | 0.0 | 0.29 | 0.0 | 0.02 | 0.01 | 0.0 | 0.0 | 0.0 | 0.12 | 0.0 | 0.0 | 0.0 | 0.0 | 0.0 | 0.0 | 0.0 | 0.10 | 260.3 |
| **GSTM3** | 0.27 | 0.15 | 7.51 | 2.29 | 2.67 | 0.37 | 0.84 | 1.27 | 1.39 | 1.08 | 1.04 | 1.67 | 0.53 | 1.05 | 20.2 | 29.2 | 1.57 | 4.30 | 8.00 | 186.4 |
| **GSTP1 rs1695** | 4.66 | 1.42 | 2.60 | 4.89 | 6.92 | 1.66 | 2.55 | 0.02 | 0.78 | 1.47 | 4.46 | 4.23 | 2.09 | 3.08 | 5.72 | 6.62 | 0.47 | 3.20 | 2.10 | 67.9 |
| **MAOB** | 0.01 | 0.03 | 0.22 | 0.40 | 1.23 | 0.03 | 0.30 | 0.0 | 0.0 | 0.02 | 0.17 | 0.0 | 0.03 | 0.0 | 0.0 | 0.02 | 0.0 | 0.10 | 0.30 | 210.7 |

Sample identifiers indicate tissue and donor information: **D** = Dermis, **E** = Epidermis, **M** = Membrane fraction, **c** = Caucasian

| **Supplemental Table 9:** Protein abundance enzymes in dermis and epidermis cytosol fractions from 17 human donors, expressed as pmol/mg protein. Sample numbers correspond to donor age | | | | | | | | | | | | | | | | | | | | |
| --- | --- | --- | --- | --- | --- | --- | --- | --- | --- | --- | --- | --- | --- | --- | --- | --- | --- | --- | --- | --- |
| **Enzyme** | **DCc24** | **DCc36** | **DCc37** | **DCc40** | **DCc43** | **DCc44** | **DCc46** | **DCc47** | **DCc49a** | **DCc49b** | **DCc50** | **DCc52** | **DCc53** | **DCc54** | **DCc62** | **DCc64** | **DCc69** | **Mean** | **SD** | **% CV** |
| **AKR1A1** | 0.10 | 0.10 | 0.10 | 0.0 | 0.10 | 0.10 | 0.0 | 0.0 | 0.0 | 0.0 | 0.10 | 0.50 | 0.10 | 0.0 | 0.0 | 0.0 | 0.0 | 0.1 | 0.1 | 147.0 |
| **AKR1B1** | 0.10 | 0.30 | 0.10 | 0.10 | 0.0 | 0.10 | 0.10 | 0.0 | 0.0 | 0.0 | 0.10 | 0.30 | 0.10 | 0.0 | 0.0 | 0.1 | 0.1 | 0.1 | 0.1 | 108.8 |
| **AKR1C1** | 0.0 | 0.0 | 0.0 | 0.0 | 0.0 | 0.0 | 0.0 | 0.0 | 0.0 | 0.0 | 0.0 | 0.10 | 0.0 | 0.3 | 0.0 | 0.0 | 0.0 | 0.0 | 0.1 | 327.8 |
| **AKR1C2** | 0.0 | 0.0 | 0.0 | 0.0 | 0.0 | 0.0 | 0.0 | 0.0 | 0.0 | 0.0 | 0.0 | 0.10 | 0.0 | 0.0 | 0.0 | 0.0 | 0.0 | 0.0 | 0.0 | 253.6 |
| **AKR7A2** | 0.0 | 0.0 | 0.0 | 0.0 | 0.0 | 0.0 | 0.0 | 0.0 | 0.0 | 0.0 | 0.0 | 0.10 | 0.0 | 0.0 | 0.0 | 0.0 | 0.0 | 0.0 | 0.0 | 254.2 |
| **ALDH16A1** | 0.0 | 0.0 | 0.0 | 0.0 | 0.0 | 0.0 | 0.0 | 0.0 | 0.0 | 0.0 | 0.0 | 0.10 | 0.0 | 0.0 | 0.0 | 0.0 | 0.0 | 0.0 | 0.0 | 321.1 |
| **ALDH1A1** | 5.60 | 7.10 | 3.30 | 4.30 | 2.90 | 2.20 | 2.10 | 2.20 | 5.40 | 4.40 | 3.30 | 9.20 | 2.90 | 5.3 | 9.4 | 4.6 | 4.4 | 4.6 | 2.2 | 48.1 |
| **ALDH1A2** | 0.0 | 0.0 | 0.0 | 0.0 | 0.0 | 0.40 | 0.40 | 0.0 | 0.0 | 0.20 | 0.10 | 0.10 | 0.20 | 0.0 | 0.0 | 0.0 | 0.1 | 0.1 | 0.1 | 153.8 |
| **ALDH1B1** | 0.0 | 1.50 | 0.30 | 2.00 | 0.70 | 0.70 | 1.40 | 0.0 | 0.0 | 0.10 | 1.10 | 1.10 | 1.60 | 0.0 | 0.1 | 0.2 | 1.4 | 0.7 | 0.7 | 95.2 |
| **ALDH2** | 4.10 | 5.20 | 4.60 | 8.00 | 4.60 | 2.00 | 3.80 | 2.40 | 12.8 | 3.60 | 3.30 | 5.40 | 5.40 | 4.70 | 7.20 | 3.40 | 4.60 | 5.00 | 2.5 | 49.8 |
| **ALDH3A1** | 1.60 | 1.00 | 0.40 | 0.50 | 0.40 | 0.60 | 0.60 | 2.10 | 0.20 | 0.80 | 0.30 | 1.70 | 0.70 | 2.00 | 0.70 | 3.20 | 1.20 | 1.10 | 0.80 | 76.9 |
| **ALDH4A1** | 0.0 | 0.0 | 0.10 | 0.0 | 0.0 | 0.0 | 0.0 | 0.0 | 0.0 | 0.0 | 0.0 | 0.0 | 0.0 | 0.0 | 0.0 | 0.0 | 0.0 | 0.0 | 0.0 | 178.2 |
| **ALDH7A1** | 13.4 | 3.60 | 3.10 | 3.60 | 5.00 | 9.50 | 11.9 | 11.5 | 2.30 | 4.90 | 2.90 | 6.40 | 5.20 | 7.50 | 7.10 | 2.70 | 5.90 | 6.30 | 3.50 | 55.1 |
| **ALDH9A1** | 4.30 | 4.30 | 1.50 | 1.90 | 8.00 | 1.80 | 2.40 | 1.20 | 5.80 | 2.40 | 3.30 | 7.30 | 2.10 | 3.60 | 5.20 | 2.50 | 4.50 | 3.70 | 2.00 | 54.9 |
| **AOC3** | 0.30 | 0.60 | 0.50 | 2.40 | 0.70 | 1.50 | 1.50 | 1.00 | 0.60 | 1.30 | 0.40 | 3.70 | 0.30 | 2.10 | 0.80 | 0.80 | 1.30 | 1.20 | 0.90 | 77.3 |
| **AOX1** | 0.0 | 0.0 | 0.0 | 0.70 | 0.30 | 0.0 | 0.0 | 0.0 | 0.0 | 0.0 | 0.10 | 0.30 | 0.10 | 0.20 | 0.0 | 0.0 | 0.0 | 0.10 | 0.20 | 195.0 |
| **BCHE** | 0.10 | 0.60 | 0.20 | 2.0 | 0.60 | 2.10 | 2.70 | 0.0 | 0.0 | 0.20 | 0.50 | 0.60 | 0.80 | 0.10 | 0.20 | 2.00 | 0.60 | 0.80 | 0.90 | 110.8 |
| **CES1** | 7.50 | 9.70 | 13.3 | 11.4 | 21.1 | 7.40 | 12.7 | 8.10 | 5.70 | 17.5 | 5.30 | 8.00 | 6.90 | 7.20 | 23.6 | 15.7 | 13.7 | 11.5 | 5.40 | 47.5 |
| **EPHX1** | 0.0 | 0.0 | 0.10 | 0.0 | 1.00 | 0.10 | 0.10 | 0.0 | 0.0 | 0.20 | 0.30 | 1.10 | 0.10 | 1.00 | 0.20 | 0.0 | 0.0 | 0.20 | 0.40 | 160.5 |
| **ESD** | 1.10 | 1.40 | 1.10 | 1.00 | 0.80 | 0.70 | 0.60 | 0.0 | 0.0 | 0.50 | 1.20 | 2.30 | 1.00 | 0.50 | 0.70 | 0.70 | 0.90 | 0.80 | 0.50 | 62.8 |
| **GGT5** | 0.0 | 0.0 | 0.0 | 0.0 | 0.0 | 0.0 | 0.0 | 0.0 | 0.0 | 0.0 | 0.0 | 0.10 | 0.0 | 0.10 | 0.0 | 0.0 | 0.0 | 0.0 | 0.0 | 172.9 |
| **GPX1** | 0.0 | 0.0 | 0.0 | 0.0 | 0.0 | 0.0 | 0.0 | 0.0 | 0.0 | 0.0 | 0.0 | 0.10 | 0.0 | 0.0 | 0.0 | 0.0 | 0.0 | 0.0 | 0.0 | 412.3 |
| **GPX3** | 0.20 | 0.40 | 0.10 | 2.60 | 0.70 | 0.50 | 0.60 | 0.20 | 0.0 | 0.30 | 1.20 | 10.8 | 0.50 | 1.10 | 0.10 | 0.0 | 0.20 | 1.1 | 2.6 | 225.2 |
| **GPX7** | 0.0 | 1.30 | 0.0 | 0.0 | 2.60 | 0.0 | 0.0 | 0.0 | 0.0 | 0.0 | 1.30 | 0.10 | 0.0 | 0.0 | 0.0 | 0.0 | 0.0 | 0.30 | 0.70 | 232.5 |
| **GSTM1** | 4.80 | 5.50 | 0.50 | 2.70 | 0.10 | 2.10 | 1.80 | 0.0 | 0.20 | 0.30 | 2.00 | 0.60 | 0.40 | 2.60 | 26.6 | 1.90 | 3.50 | 3.30 | 6.20 | 189.9 |
| **GSTM2** | 0.20 | 0.30 | 0.50 | 0.10 | 0.0 | 0.20 | 0.10 | 0.0 | 0.0 | 0.20 | 0.10 | 0.30 | 0.10 | 0.0 | 2.80 | 0.0 | 0.10 | 0.30 | 0.70 | 224.9 |
| **GSTM3** | 0.10 | 0.90 | 0.10 | 0.10 | 1.00 | 0.0 | 0.0 | 0.0 | 0.0 | 0.10 | 0.10 | 0.60 | 0.0 | 0.0 | 0.40 | 0.10 | 0.10 | 0.20 | 0.30 | 150.2 |
| **GSTP1** | 13.2 | 12.9 | 2.50 | 13.9 | 8.40 | 4.30 | 7.20 | 5.10 | 17.3 | 4.30 | 6.90 | 13.4 | 14.9 | 19.2 | 8.20 | 1.90 | 9.80 | 9.60 | 5.20 | 54.6 |
| **GSTP1 rs1695** | 0.0 | 2.20 | 6.10 | 0.0 | 1.90 | 0.70 | 1.60 | 0.0 | 0.0 | 1.00 | 0.0 | 0.20 | 0.50 | 0.0 | 2.70 | 5.10 | 0.70 | 1.30 | 1.80 | 137.3 |
| **LTA4H** | 2.30 | 2.50 | 0.40 | 2.50 | 0.70 | 0.20 | 0.30 | 0.0 | 0.0 | 2.40 | 0.50 | 2.00 | 0.80 | 0.50 | 2.80 | 1.90 | 0.70 | 1.20 | 1.00 | 85.0 |
| **Enzyme** | **ECc24** | **ECc36** | **ECc37** | **ECc40** | **ECc43** | **ECc44** | **ECc46** | **ECc47** | **ECc49a** | **ECc49b** | **ECc50** | **ECc52** | **ECc53** | **ECc54** | **ECc62** | **ECc64** | **ECc69** | **Mean** | **SD** | **% CV** |
| **AKR1A1** | 0.30 | 0.3 | 0.0 | 0.0 | 0.1 | 0.2 | 0.1 | 0.0 | 0.0 | 1.5 | 0.1 | 0.0 | 0.1 | 0.0 | 0.0 | 0.0 | 0.1 | 0.2 | 0.4 | 207.3 |
| **AKR1B1** | 0.30 | 0.4 | 0.0 | 0.1 | 0.1 | 0.1 | 0.2 | 0.0 | 0.0 | 0.2 | 0.1 | 0.0 | 0.1 | 0.0 | 0.0 | 0.0 | 0.2 | 0.1 | 0.1 | 113.9 |
| **AKR1C2** | 0.0 | 0.0 | 0.0 | 0.0 | 0.0 | 0.0 | 0.0 | 0.0 | 0.0 | 0.2 | 0.0 | 0.0 | 0.0 | 0.0 | 0.0 | 0.0 | 0.0 | 0.0 | 0.0 | 229.1 |
| **AKR1C3** | 0.10 | 0.1 | 0.0 | 0.0 | 0.0 | 0.0 | 0.0 | 0.0 | 0.0 | 0.1 | 0.0 | 0.0 | 0.0 | 0.0 | 0.0 | 0.0 | 0.0 | 0.0 | 0.0 | 148.0 |
| **ALDH1A1** | 6.70 | 9.8 | 0.0 | 7.0 | 4.8 | 3.2 | 4.2 | 0.0 | 0.0 | 7.0 | 5.7 | 0.0 | 5.0 | 0.0 | 0.0 | 0.0 | 9.5 | 3.7 | 3.6 | 96.8 |
| **ALDH1A2** | 0.10 | 0.2 | 0.0 | 0.5 | 0.2 | 0.6 | 0.9 | 0.0 | 0.0 | 0.0 | 0.1 | 0.0 | 0.7 | 0.0 | 0.0 | 0.0 | 0.2 | 0.2 | 0.3 | 148.1 |
| **ALDH1B1** | 3.80 | 6.0 | 0.0 | 6.7 | 4.0 | 1.6 | 2.8 | 0.0 | 0.0 | 1.7 | 3.8 | 0.0 | 10.4 | 0.0 | 0.0 | 0.0 | 4.1 | 2.6 | 3.0 | 114.1 |
| **ALDH2** | 13.3 | 32.9 | 0.0 | 21.7 | 17.1 | 6.6 | 11.2 | 0.0 | 0.0 | 5.7 | 13.3 | 0.0 | 27.4 | 0.0 | 0.0 | 0.0 | 16.1 | 9.7 | 10.6 | 109.2 |
| **ALDH3A1** | 2.40 | 4.2 | 0.0 | 2.4 | 2.9 | 2.0 | 2.1 | 0.0 | 0.0 | 3.8 | 1.4 | 0.0 | 3.8 | 0.0 | 0.0 | 0.0 | 3.2 | 1.7 | 1.6 | 95.4 |
| **ALDH3A2** | 0.0 | 0.0 | 0.0 | 0.0 | 0.0 | 0.0 | 0.0 | 0.0 | 0.0 | 0.0 | 0.0 | 0.0 | 0.0 | 0.0 | 0.0 | 0.0 | 0.0 | 0.0 | 0.0 | 412.3 |
| **ALDH4A1** | 0.20 | 0.0 | 0.0 | 0.2 | 0.1 | 0.7 | 1.3 | 0.0 | 0.0 | 0.1 | 0.1 | 0.0 | 0.5 | 0.0 | 0.0 | 0.0 | 0.0 | 0.2 | 0.3 | 177.0 |
| **ALDH7A1** | 6.80 | 9.7 | 0.0 | 14.2 | 7.8 | 21.3 | 30.5 | 0.0 | 0.0 | 8.0 | 5.9 | 0.0 | 11.8 | 0.0 | 0.0 | 0.0 | 14.5 | 7.7 | 8.8 | 114.4 |
| **ALDH9A1** | 9.70 | 5.8 | 0.0 | 6.4 | 9.1 | 3.7 | 3.1 | 0.0 | 0.0 | 1.9 | 8.5 | 0.0 | 6.4 | 0.0 | 0.0 | 0.0 | 8.0 | 3.7 | 3.8 | 101.9 |
| **AOC3** | 0.10 | 0.2 | 0.0 | 0.2 | 0.2 | 0.4 | 1.8 | 0.0 | 0.0 | 0.0 | 0.1 | 0.0 | 0.2 | 0.0 | 0.0 | 0.0 | 0.2 | 0.2 | 0.4 | 212.4 |
| **BCHE** | 0.10 | 0.1 | 0.0 | 0.2 | 1.1 | 0.4 | 0.4 | 0.0 | 0.0 | 0.1 | 0.1 | 0.0 | 1.2 | 0.0 | 0.0 | 0.0 | 0.3 | 0.2 | 0.4 | 163.4 |
| **CES1 rs2307240** | 8.50 | 16.6 | 0.90 | 10.0 | 10.0 | 11.2 | 23.1 | 0.0 | 0.0 | 3.5 | 6.6 | 0.0 | 13.8 | 0.0 | 1.2 | 1.1 | 20.8 | 7.5 | 7.6 | 101.8 |
| **CYP20A1** | 0.0 | 0.0 | 0.0 | 0.0 | 0.0 | 0.0 | 0.0 | 0.0 | 0.0 | 0.0 | 0.0 | 0.0 | 0.0 | 0.0 | 0.0 | 0.0 | 0.0 | 0.0 | 0.0 | 185.8 |
| **EPHX2** | 0.0 | 0.0 | 0.0 | 0.0 | 0.0 | 0.0 | 0.0 | 0.0 | 0.0 | 0.0 | 0.0 | 0.0 | 0.0 | 0.0 | 0.0 | 0.0 | 0.0 | 0.0 | 0.0 | 185.8 |
| **ESD** | 1.20 | 1.6 | 0.0 | 1.8 | 0.9 | 1.4 | 1.2 | 0.0 | 0.0 | 3.0 | 1.5 | 0.0 | 1.9 | 0.0 | 0.0 | 0.0 | 1.1 | 0.9 | 0.9 | 98.7 |
| **GSTM1** | 2.00 | 9.9 | 0.0 | 3.0 | 1.1 | 4.2 | 3.9 | 0.0 | 0.0 | 2.4 | 3.6 | 0.0 | 0.3 | 0.0 | 0.0 | 0.0 | 8.4 | 2.3 | 3.0 | 132.2 |
| **GSTM2** | 0.70 | 1.1 | 0.0 | 0.5 | 1.1 | 1.0 | 1.2 | 0.0 | 0.0 | 0.9 | 0.5 | 0.0 | 0.3 | 0.0 | 0.0 | 0.0 | 0.9 | 0.5 | 0.5 | 100.2 |
| **GSTM3** | 0.0 | 0.1 | 0.0 | 0.0 | 0.0 | 0.0 | 0.0 | 0.0 | 0.0 | 0.3 | 0.0 | 0.0 | 0.0 | 0.0 | 0.0 | 0.0 | 0.0 | 0.0 | 0.1 | 318.0 |
| **GSTP1** | 39.2 | 28.1 | 0.0 | 14.0 | 13.5 | 11.9 | 13.2 | 0.0 | 0.0 | 5.4 | 16.2 | 0.0 | 21.8 | 0.0 | 0.0 | 0.0 | 22.7 | 10.9 | 11.9 | 108.8 |
| **LTA4H** | 6.20 | 6.7 | 0.0 | 1.3 | 1.5 | 1.5 | 1.6 | 0.0 | 0.0 | 9.6 | 1.7 | 0.0 | 1.3 | 0.0 | 0.0 | 0.0 | 1.1 | 1.9 | 2.8 | 148.7 |

Sample identifiers indicate tissue and donor information: **D** = Dermis, **E** = Epidermis, **C** = Cytosol fraction, **c** = Caucasian

| **Supplemental Table 10:** The presence of GSTP1 and GSTP1(105 I→V) unique peptides YISLIYTNYEAGK and YVSLIYTNYEAGK respectively in four fractions of Caucasian human skin. ✓ = <10000 intensity, ✓✓ = 10000 – 100000 intensity, *= 100000 - 1000000 intensity, ** = >1000000 intensity. Note that in the epidermis and dermis cytosol the miscleaved YISLIYTNYEAGKDDYVK is also seen. The intensities are very similar to YISLIYTNYEAGK and the sum is shown here. Sample numbers correspond to donor age. | | | | | | | | | | | | | | | | | |
| --- | --- | --- | --- | --- | --- | --- | --- | --- | --- | --- | --- | --- | --- | --- | --- | --- | --- |
| **Sample** | **C24** | **C36** | **C37** | **C40** | **C43** | **C44** | **C46** | **C47** | **C49a** | **C49b** | **C50** | **C52** | **C53** | **C54** | **C62** | **C64** | **C69** |
| **EC** YISLIYTNYEAGK | ** | ** |  | ** | ** | ** | ** |  |  | ** | ** |  | ** |  |  |  | ** |
| **DC** YISLIYTNYEAGK | * | ** | ** | * | ✓✓ | ** | * | * | * | ** | ** | ** | ** | * | ** | ✓✓ | ** |
| **EM** YISLIYTNYEAGK |  |  |  |  |  |  |  |  |  |  |  |  |  |  |  |  |  |
| **DM** YISLIYTNYEAGK | * | ✓✓ | ✓ | ✓✓ | * |  | ✓✓ |  |  | ✓✓ | * |  |  |  |  | ✓ | ✓✓ |
| **EC** YVSLIYTNYEAGK |  |  |  |  |  |  |  |  |  |  |  |  |  |  |  |  |  |
| **DC** YVSLIYTNYEAGK |  | ** | ** | ✓ | ✓✓ | * | * |  |  | * | ✓✓ | * | ✓✓ |  | * | * | * |
| **EM** YVSLIYTNYEAGK | ✓ | ✓✓ | ✓ | * | * | ✓ | ✓✓ |  |  |  | ✓✓ |  | ✓ |  |  | ✓ | ✓ |
| **DM** YVSLIYTNYEAGK |  |  | ✓ |  | ✓ | ✓✓ | ✓✓ |  |  | ✓ | ✓ |  |  |  |  | ✓ | ✓✓ |
